# Music students' psychological profiles: unveiling three coping clusters using schema mode inventory

**DOI:** 10.3389/fpsyg.2025.1673100

**Published:** 2026-01-05

**Authors:** Teresa Wenhart, Horst Hildebrandt

**Affiliations:** 1 Independent Researcher, Zürich, Switzerland; 2 Department of Music, Institute of Music Research, Zurich University of the Arts, Zürich, Switzerland; 3 Swiss University Center for Music Physiology, Basel Music Academy, Basel, Switzerland

**Keywords:** musicians, mental health, schema modes, coping strategies, coaching, psychological profiles, self-talk, resilience

## Abstract

**Background and aim:**

Professional musicians face unique psychological demands leading to elevated rates of mental health problems, including depression, anxiety, and performance anxiety, as well as stress-related disorders. These difficulties are associated with perfectionism, adverse experiences, and maladaptive coping strategies. While schema modes—recurring emotion, cognitive, and behavioral patterns triggered by early maladaptive schemas—are well-studied in clinical populations, their role in musicians remains unexplored. This study explores schema-mode presence in music students to evaluate their utility for understanding psychological vulnerability and coping.

**Methods:**

In total, 46 music students from Zurich University of the Arts and Basel Music Academy completed an online survey assessing schema modes (short Schema Mode Inventory), musician-specific coping (HIL scale), and self-talk and complaints related to music-making via open-ended questions. Analysis included comparisons of effect sizes with normative summary data from healthy controls and clinical patients, inter-correlations between schema-modes and coping, cluster analysis identifying psychological profiles, and qualitative content analysis.

**Results:**

Music students scored significantly higher on maladaptive schema-modes vs. non-clinical controls, indicating greater emotional coping difficulties and reduced adaptive resources. Coping capacity correlated negatively with maladaptive modes and positively with the Healthy Adult mode. Scores overlapped with those of Axis I patients but differed from Axis II patients, suggesting intermediate clinical characteristics. Cluster analysis revealed three distinct profiles: “Balanced Musicians” (resilient cluster with high Healthy Adult and Happy Child modes and effective coping), “Vulnerable Musicians” (high-risk cluster with intense emotional child modes and frequent maladaptive parent/coping modes), and “Compensating Musicians” (at-risk cluster with intermediate scores and overcompensating strategies combining functional and maladaptive modes).

**Conclusion:**

Schema modes appear central to musicians' mental health and coping, highlighting psychological profile heterogeneity among music students. Schema-focused interventions targeting maladaptive modes may enhance resilience and mental health in this population. This approach offers a promising clinical framework for supporting musicians' wellbeing.

## 1 Introduction

Music making and music listening have been found to positively impact wellbeing (Altenmüller and Schlaug, 2012), e.g., by reducing stress responses (Iyendo, 2016; Kreutz et al., 2012; Thoma et al., 2013) and pain perception (Iyendo, 2016; Spintge and Droh, 2012), and by exerting positive effects on the immune response (Kreutz et al., 2012; Thoma et al., 2013; Kuhn, 2002; Kreutz et al., 2004), brain plasticity (Altenmüller and Schlaug, 2012; Münte et al., 2002; Moreno et al., 2009; Reybrouck et al., 2018), neurorehabilitation (Sihvonen et al., 2017; Altenmüller and Schlaug, 2013), and healthy aging (James et al., 2020; Wan and Schlaug, 2010; Böttcher et al., 2022; Worschech et al., 2025). However, in professional musicians, as in athletes, the high physical and mental demands of professionally mastering a musical instrument and performing in the industry have been linked to stress and occupation-specific illnesses of musicians. Fields that have long been established for athletes—sports physiology, sports psychology, and sports medicine—have now evolved into a separate specialist field for the needs of professional musicians under the name music physiology and musicians' medicine. In the context of increasing technical and performance demands on professional musicians and heightened competition in the global music industry, research has increasingly focused on the physiological and psychological conditions of music-making (Vervainioti and Alexopoulos, 2015; Middlestadt and Fishbein, 1988) and the pathophysiological mechanisms underlying occupational health problems in musicians.

The most common complaints among musicians include musculoskeletal imbalances and pain disorders (Kenny and Ackermann, 2015; Silva et al., 2015; Leaver et al., 2011; Ackermann et al., 2012), various stress-related psychosomatic disorders (Kenny et al., 2014; Spahn, 2018; Hildebrandt et al., 2012), excessive music performance anxiety (Kenny et al., 2014; Hildebrandt et al., 2012; Spahn et al., 2021a,b, 2016; Kenny, 2011), and musicians' dystonia (Altenmüller and Jabusch, 2009). Common contributing factors include an imbalance between demands and coping resources, as well as professional and private stress situations (Vervainioti and Alexopoulos, 2015; Middlestadt and Fishbein, 1988; Kenny et al., 2014; Loveday et al., 2023; Sataloff et al., 1998; Schneider et al., 2021; Kenny and Ackermann, 2016; Musgrave, 2023a,b). Consistent with the vulnerability-stress model (Lazarus and Folkman, 1984; Folkman et al., 1986), both musculoskeletal disorders and musicians' dystonia have been linked to psychological factors such as perfectionism (Alpheis et al., 2022; Ioannou et al., 2016; Borngräber and Schmidt, 2020; Ioannou and Altenmüller, 2014), anxiety (Borngräber and Schmidt, 2020; Jabusch et al., 2004; Jabusch and Altenmüller, 2004), and psychosocial stress (Middlestadt and Fishbein, 1988; Ioannou et al., 2016; Alpheis et al., 2023), as well as to traumatic childhood experiences (Schneider et al., 2021; Alpheis et al., 2022). Research indicates that musicians experience higher rates of mental health issues compared to the general population. Kenny et al. (2014) found high prevalence rates of affective disorders among Australian professional orchestra musicians, including social phobia (33%), post-traumatic stress disorder (PTSD; 22%), and depression (32%). Research by Vaag et al. (2016a,b) found prevalence rates of 20.1% for symptoms of depression and 14.7% for symptoms of anxiety, as well as increased use of psychotherapy and psychotropic medication and a higher prevalence of impaired sleep (Vaag et al., 2016c) among Norwegian professional musicians. Solo artists, lead musicians, and internationally touring performers exhibit the highest risks for mental health issues (Loveday et al., 2023; Newman et al., 2022).

With regard to music performance anxiety (MPA), Spahn et al. (2021b) found three subgroups of musicians, which are distinguished from each other by symptom severity and the availability of self-efficacy and functional coping strategies. While half of the sample exhibited only a few symptoms of MPA [Type I MPA, common stage fright or “Lampenfieber” (Spahn et al., 2021b)], approximately one quarter showed high symptoms at the beginning of the performance [Type II MPA (Spahn et al., 2021b)] vs. increasing symptoms during performance [Type III MPA (Spahn et al., 2021b)]. The latter group exhibited the lowest values of self-efficacy and adaptive coping and the lowest self-assessment of musical quality (Spahn et al., 2021b). Given the relationship between symptom severity and maladaptive coping (Spahn et al., 2021b), we hypothesize that these individuals tend to overly focus on or become triggered by errors and/or physical symptoms during performance. They may become automatically trapped in negative inner self-talk, leading to a vicious cycle of increased symptoms and declining performance quality. This phenomenon may occur not only during public performances but also in lessons or chamber music settings, e.g., as a reaction to criticism or by being observed and judged by others (so-called triggers).

Schema therapy (also known as schema-focused therapy, SFT), with its focus on early maladaptive schemas (EMS), appears particularly well-suited to address these challenges. For readers unfamiliar with this approach, we provide a brief overview of schema therapy. Schema therapy is an integrative psychological treatment approach developed by Young et al. (2006) that aims to help individuals understand and change long-standing, deeply ingrained negative patterns of thinking, feeling, and behaving. These patterns typically originated in childhood and continue to cause problems in adult life. Known as early maladaptive schemas, they are core beliefs or emotional themes that act as a lens through which individuals view themselves, others, and the world. When these schemas are triggered by current life events, they can lead to intense emotional reactions and unhelpful coping strategies. Schema therapy is particularly effective for addressing complex psychological challenges because it combines elements from cognitive-behavioral therapy, attachment theory, psychodynamic concepts, and Gestalt therapy. At its core, schema therapy focuses on identifying and modifying early maladaptive schemas, which are self-defeating emotional and cognitive patterns that begin early in development and repeat throughout life. Young et al. (2006) identified 18 early maladaptive schemas that are grouped into five broad domains based on unmet emotional needs in childhood: (1) Disconnection and Rejection, (2) Impaired Autonomy and Performance, (3) Impaired Limits, (4) Other-Directedness, and (5) Overvigilance and Inhibition. Beyond schemas, schema therapy also utilizes the concept of schema-modes, which are moment-to-moment emotional and cognitive states and coping strategies (Young et al., 2006; Lobbestael et al., 2007). These modes represent different emotional and behavioral states that are activated in response to specific situations, often triggered by emotional events related to our personal schemas. Individuals can rapidly shift between these modes (Young et al., 2006; Lobbestael et al., 2007). Young et al. (2006) described four main types of schema modes (see Table 1 for an overview and detailed description):

1) *Child modes*: Represent innate, universal emotional states from childhood (e.g., vulnerable child, angry child, and impulsive child);
2) *Maladaptive coping modes*: Represent strategies for coping with schema activation (e.g., detached protector, compliant surrenderer, and self-aggrandizer);
3) *Internalized parent modes*: Represent internalized voices of parents or authority figures (e.g., punitive parent and demanding parent);
4) *Healthy adult mode*: Represents the integrated, functional aspect of the self, capable of nurturing, limit-setting, and problem-solving.

**Table 1:** Schema modes according to short SMI.

**Acronym**	**Schema mode (short SMI, Lobbestael et al., 2010)**	**Emotional and behavioral type**	**Mode category**
VC	Vulnerable child	Sadness, shame, fear, feeling fundamentally inadequate & excluded, loneliness	Maladaptive child modes
AC	Angry child	Anger, e.g., in case of abundance, lack of freedom/independence, revenge, feeling unfairly treated/cheated	
EC	Enraged child	Rage, out of control anger with intense impulses to destroy things/hurt other people, threatening other people	
IC	Impulsive child	Impatience, lack of self-control	
UC	Undisciplined child	Lack of self-control, dismissing boundaries and rules, procrastination, boring tasks	
HC	Happy child mode	Curiosity, happiness, fun, feeling safe, loved, and accepted	Happy child mode
CS	Compliant surrender	“Freeze”—response, people pleasing, passivity, avoiding conflict, social chameleon, not expressing own needs, underdog	Maladaptive coping modes (“Protectors” against unpleasant child modes/emotions and inner critic)
DPT	Detached protector	“Flight”—response; procrastination, resignation, not-responding/interacting, dissociation, emotional numbness, emotionally detached	
DSS	Detached self-soother	“Flight”—response; distracting and addictive behavior (social media, drugs, work, and gaming), rumination, daydreaming	
SA	Self-aggrandizer	“Fight”—response; seeking attention of others, ambition to always be Nr. 1, neglecting other people's feelings and needs, need to control other people	
BA	Bully & attack	“Fight”—response; dominant behavior, belittling & bullying others	
PP	Punishing parent	Denying oneself pleasures, self-harming behavior, feeling of being a bad person, angry at oneself	Maladaptive inner critic
DP	Demanding parent	Trying hard to do things “right,” high own standards, sacrificing health and wellbeing, perfectionism, constant self-pressure to achieve	
HA	Healthy adult	Feeling to be a good person, self-sufficient, self-structured, healthy boundaries (self and others), learning mindset, optimistic, adequate emotional regulation	Adaptive, reflected and flexible adult mode

14 Schema modes according to the factor structure of short SMI (Lobbestael et al., 2010). Column “emotional and behavioral response” describes typical feelings, beliefs and behavior associated with the mode and evaluated by the respective test items.

To illustrate these dynamic internal states, consider a music student receiving critical feedback from a teacher. If this feedback triggers an underlying **Defectiveness/Shame schema**, the student might initially experience a **Vulnerable Child mode**, feeling deeply hurt, inadequate, and believing, “I am fundamentally flawed and exposed.” This intense emotional pain can then immediately activate various coping responses. One common reaction is a **Detached Protector mode** (avoiding mode, “Flight”), leading them to emotionally disconnect and appear indifferent to the feedback as a defense. Alternatively, they might shift into a maladaptive coping mode, such as the **Self-Aggrandizer mode** (overcompensating mode, “Fight”), where they might dismiss the criticism, become defensive, or even undermine the teacher's authority to protect their threatened self-esteem. Simultaneously, an internal **Punitive Parent mode** might emerge, with self-critical thoughts like, “You are a complete failure; this proves you don't belong here,” intensifying the shame. This complex interplay of modes is further influenced by how criticism is delivered. Harsh or shaming feedback from the teacher can exacerbate the student's **Vulnerable Child** and **Punitive Parent modes**, while constructive and supportive criticism might facilitate a shift toward a more adaptive response. Additionally, the teacher's own schemas (e.g., **Unrelenting Standards**) can unconsciously shape their critical approach, adding another layer to the interaction. This example illustrates how various schema modes often co-activate, creating a complex internal landscape that influences behavioral responses in challenging situations. In contrast, a student operating from a balanced Healthy Adult mode would be able to process the feedback constructively, acknowledge areas for improvement without self-condemnation, and engage in adaptive problem-solving (adapted from Wenhart, 2024).

Schema modes provide a useful framework for conceptualizing the emotional, cognitive, and behavioral issues of a client or patient. Schema-focused therapy—including coaching applications (McCormick et al., 2022; Migge, 2014)—aims to balance schema modes and foster adaptive coping strategies in response to emotional triggers.

Research on schemas and schema-focused therapy has led to the development of various questionnaires to assess (1) early maladaptive schemas (Bach et al., 2015; Kriston et al., 2013; Phillips et al., 2019; Rijkeboer, 2012) and (2) schema modes (Lobbestael et al., 2010; Simpson et al., 2018; Harris, 2010). Most available studies have assessed schema mode profiles in samples with personality disorders (Lobbestael et al., 2008; Arntz et al., 2005; Jacob and Arntz, 2013; Lobbestael et al., 2005). A systematic review (Masley et al., 2012) found medium-to-large effect sizes for schema therapy interventions, particularly for personality disorders. Randomized trials comparing schema therapy for personality disorders to psychodynamic (Giesen-Bloo et al., 2006) and to clarification-oriented therapy (Bamelis et al., 2014) demonstrated significantly greater recovery in the schema-therapy group for borderline, anxious, paranoid, histrionic, and narcissistic personality disorders. Furthermore, schema modes have been investigated in eating disorders (Talbot et al., 2015), obsessive–compulsive disorders (Tenore et al., 2018; Basile et al., 2017; Gross et al., 2012; Peeters et al., 2022), and affective and anxiety disorders (Peeters et al., 2022; Hawke and Provencher, 2011; Renner et al., 2013; Penney and Norton, 2022).

### 1.1 Research gap and hypothesis

To the best of our knowledge, no previous study has investigated the presence and relative expression of schemas or schema modes in musicians. Given its focus on early maladaptive schemas, personality development, and multifaceted intervention strategies, schema-focused therapy appears particularly well-suited to address the above-mentioned challenges and vulnerabilities of professional musicians and music students.

The present study investigates schema modes in music students and their relation to the results of non-clinical, Axis I and Axis II disorders [using the short Schema Mode Inventory, short SMI (Lobbestael et al., 2010)] and coping with professional demands of musicians (HIL Scale, Hildebrandt, 2004). We chose to investigate schema modes using the short SMI due to the availability of normative comparison data in the validation study (Lobbestael et al., 2010) and its clinical applicability. Schema therapy and schema coaching primarily work with modes rather than underlying schemas, making this instrument particularly suitable for our purposes. The short SMI (Lobbestael et al., 2010) is a widely cited instrument designed to assess schema modes—temporary states of emotions, cognitions, and behaviors associated with underlying schemas. This instrument provides normative group-level data for healthy controls as well as clinical samples, including Axis I and Axis II patients. While most other studies focus on individual psychiatric disorders, the short SMI provides data on clustered disorder groups within a single publication. This is particularly valuable because our study is the first investigation of schema modes or schema-related psychology in musicians. Comparing musicians to a single disorder would be too narrow, while comparing across multiple studies with varying methodologies would introduce excessive heterogeneity. Although the Axis I and II classification is based on DSM-IV (Axis I: clinical disorders such as depression and anxiety; Axis II: personality disorders), we selected this study due to its large sample sizes and the breadth of clinical groups represented, providing a valuable reference for interpreting the severity and patterns of maladaptive and coping schema modes in our sample.

Given existing research on music performance anxiety (MPA), substance use in musicians, and mediating factors including childhood trauma, perfectionism, anxiety, and depression in the development of musicians' health problems, we hypothesize that, compared to non-clinical controls, music students will demonstrate:

- Elevated vulnerable child mode scores
- Higher self-soothing and overcompensating coping modes (particularly perfectionism-related patterns)
- Increased internalized demanding and punitive parent mode scores
- Reduced healthy adult mode scores.

## 2 Methods

### 2.1 Participants and procedure

Music students enrolled in musical performance and/or music pedagogy studies at Zurich University of Arts and Basel Music Academy were recruited for the online study “Fostering Motivation and Self-Competence.” Exchange students were eligible to participate. Participants were recruited via email distribution lists and verbal announcements in music physiology classes. As compensation, students were offered individualized feedback on their questionnaire results (20 of 46 participants signed up for the offer) and a group workshop on healthy coping strategies. The workshop took place at the end of January 2025, 2 months after the completion of data collection.

While study materials used accessible, resource-oriented language, avoiding clinical terms such as “psychological,” “depression,” or ” anxiety” to minimize stigma and selection bias, participation was restricted to individuals without current diagnosed psychological or neurological disorders. This fundamental exclusion criterion was included in the consent form, and participants were required to review and confirm their eligibility before providing written consent.

Additionally, participants who had completed their primary music education (master's degree level) were excluded, including those enrolled in continuing education programs or working primarily as professional musicians. This exclusion criterion was enforced through a screening question at the beginning of the questionnaire. Participants who reported they were no longer students were directed to the final thank-you page.

In total, *N* = 46 music students (29 female, 14 male, 3 preferred not to disclose gender) with a mean age = 24.9 years (SD = 4.26, range 18 −40) of 18 nationalities participated in the study. In total, 23 students were enrolled at Zurich University of the Arts, *N* = 15 at Basel Music Academy, and *N* = 7 at other universities or preferred not to say. *N* = 24 studied music performance (bachelor or master), *N* = 8 music pedagogy, and *N* = 13 various other music performance or pedagogy subjects (e.g., music for schools, precollege, specialized education, or creative music course programs). The sample consisted of 9 pianists, 15 string players, 7 woodwind players, and 5 singers. A total of 10 participants played other instruments (represented only once in the sample) or preferred not to disclose their instrument to maintain anonymity (see Section 2.2). Participants rated their English proficiency on a 0–100 scale (0 = no understanding, 100 = native speaker), with scores uniformly distributed between a range of 52–100.

The online survey was set up and conducted via SosciSurvey (https://www.soscisurvey.de). Data processing complied with the EU General Data Protection Regulation through an agreement between SosciSurvey and the University of Zurich as data controller and processor. The study was approved by the local cantonal ethics committee (BASEC-Nr.: Req-2024-00757).

Of 115 individuals who accessed the survey, 47 completed it. One additional participant was manually excluded post-completion due to not meeting student eligibility criteria, resulting in a final sample of 46 participants. An additional 69 individuals started but did not complete the questionnaire. Of these, 13 declined consent, 2 were excluded due to not being music students, and 53 discontinued participation during the questionnaire. The completion rate in our study was approximately 41%.

### 2.2 Questionnaire material

Sociodemographic items assessed age, gender, nationality (voluntary), and self-rated English proficiency. Music-related items encompassed main instrument (voluntary), music school (voluntary), status of musicianship (precollege, student at music university, teacher, employed, and self-employed), past and current study programs (precollege, bachelor, master, and continuing education) and musical profile (pedagogy, performance, classical, jazz/rock/pop, school music, and theory/composition/sound design). Items that could compromise anonymity (e.g., due to small class sizes in specific instrument–institution combinations) were optional. For this analysis, we included only students currently enrolled in precollege, bachelor's, or master's programs in music performance or pedagogy. Additional musical training and background information were collected to provide contextual data. Participants also reported whether they experienced primarily physical complaints, psychological complaints, both types, or neither related to music-making.

#### 2.2.1 Short schema mode-inventory (short SMI)

The short Schema Mode Inventory (short SMI, Lobbestael et al., 2010) is a brief version of the Schema Mode Inventory (SMI, Young et al., 2007) and consists of 118 items, compared to 270 items in the original version. The questionnaire has a 14-factor structure (i.e., 14 schema modes; see Table 1) with acceptable internal consistency (Cronbach's α from 0.79 to 0.96) and adequate test–retest reliability. The inventory was developed to provide a shorter questionnaire for assessing schema modes in research and clinical applications and was tested on 319 non-clinical controls, 136 patients with Axis I disorders, and 236 patients with Axis II disorders (total sample: *N* = 863, 57.1% female, mean age = 34 years, *SD* = 11.80, range 18–70). Comparisons with the normative and clinical groups (see Section 3) were based on the published summary data reported in the short SMI validation study, as raw data were not available. Moreover, the questionnaire showed moderate construct validity compared with several existing scales such as *Temperament and Character Inventory* (TCI; Cloninger et al., 1994), *Irrational Belief Inventory* (IBI; Timmerman et al., 1993), *State-Trait Anger Scale* (STAS; Spielberger et al., 1983), *Childhood Trauma Questionnaire* (CTQ; Bernstein et al., 1998), *Loneliness Scale* (LS; De Jong-Gierveld and Van Tilburg, 1999)*, Relationship Scales Questionnaire* (RSQ; Griffin and Bartholomew, 1994), *Utrecht Coping List* (UCL; Schreurs et al., 1993), and *Personality Disorder Belief Questionnaire* (PDBQ Narcissism subscale; Dreessen and Arntz, 1995).

#### 2.2.2 Coping with work as a musician (HIL-scale)

The HIL Scale (Hildebrandt, 2004) assesses coping with work as a musician through seven items covering the following topics: (1) satisfaction with success at work, (2) confidence in stage situations, (3) satisfaction with breathing while playing, (4) satisfaction with posture while playing, (5) satisfaction with movements while playing, (6) symptoms in the context of music-making, and (7) feeling capable of handling one's studies or profession. Responses are recorded on a six-point scale (1 = *fully applies*, 6 = *does not apply at all*). After reversing the scores of all items (except for HIL item 6 regarding complaints), high total scores indicate good coping (maximum score = 42). The HIL Scale was tested on a sample of 68 musicians and has also been applied to 38 and 105 first-year music students and 29 music teachers. Cronbach's alpha values were 0.84, 0.73, and 0.78, indicating satisfactory reliability. Although no general population norms exist for the HIL, previous studies with music students provide reference values (compare Supplementary Table 1): e.g., first-year students (*N* = 105) scored on average 31.7–31.9 (SD = 0.45) across two time points (beginning and end of the academic year), while a similar study on first-year students (*N* = 38; Hildebrandt, 2004) reported average scores of 33.33 (SD = 4.39) and 33.06 (SD = 4.62), respectively. In another study (Hildebrandt, 2004), music teachers and advanced music students scored lower before an intervention and improved after [teachers: 26.8 (SD = 5.16) vs. 30.0 (SD = 4.78); music students: 24.6 (SD = 5.7) vs. 28.7 (SD = 5.4)]. These values suggest that scores around 30 can be interpreted as reflecting moderate to good coping, showing that participants manage the demands of musical performance and study fairly well, though there may still be room for improvement compared to the highest-performing student samples.

#### 2.2.3 Open questions: inner self-talk and complaints related to making music

The following optional open response questions were added to gain a complete understanding of the participants' adaptive and maladaptive inner self-talk, as well as other social, psychological, or physical issues:

1) If you have complaints related to music-making, what are they?
2) If you have/have had problems with a teacher or orchestra, or chamber music partner, currently or in the past, what problems were they?
3) What are typical thoughts (positive and negative) you have in musical situations (practice, rehearsal, lesson, and stage)?

These questions were not derived from previously established instruments but were developed based on our clinical experience and exchange with other experts in the field of music physiology and psychology. Question 1 reflects a commonly used open question to assess music-related problems in addition to standardized closed questions (e.g., from the HIL scale). Question 2 was included to address frequently reported difficulties in communication or hierarchical settings such as ensembles and orchestras. Question 3 aimed to capture typical patterns of inner self-talk and thoughts, providing insights into both constructive self-guidance and potentially maladaptive patterns that may reflect inner parent or child modes. We were interested in whether these aspects, which we often encounter in clinical and educational practice, might also be reflected in the schema mode results of the sample. Since no previous research has investigated schema modes in musicians or developed a respective questionnaire, we decided to include these open, exploratory questions as an initial step.

Free responses to open-ended questions were analyzed using a two-step lexical categorization procedure. First, individual responses were translated into nominalized expressions capturing the core content of each statement. Second, these expressions were grouped into descriptive categories based on similarity of content, and the frequency of each category across participants was counted. Each participant could contribute only once per category per question. The coding was conducted by a single researcher trained in qualitative analysis. The complete coding scheme, including all categories and illustrative anonymized exemplar quotes, is provided in Supplementary Table 2 to ensure transparency and reproducibility. This approach is exploratory and descriptive, aiming to provide an overview of typical complaints, self-talk, and social or organizational issues among music students, rather than to perform an interpretive thematic analysis.

### 2.4 Statistical methods

All statistical analyses were conducted using the open-source statistical software package R (Version 4.5.0) and the R packages dplyr, tidyr, tidyverse, multcomp, Hmisc, car, effsize, psych, sjstats, and stats. Plots were created with the R package ggplot2 and fmsb.

Statistical differences between the scores of our sample and the summary data (means, standard deviations, and sample sizes) of the comparison groups in Lobbestael et al. (2010) were analyzed using approximate parametric procedures (R function t.test.from.summary.data) and Cohen's d effect sizes. All within-sample analyses (e.g., group differences within the present study) were conducted using standard inferential tests based on raw data. The Shapiro-Wilk test did not reject the assumption of normality (*p* > 0.05 for all comparisons); hence, parametric tests were used for within-sample statistical analyses. Pearson correlations were used to assess the relationship between short SMI Modes and HIL Scores. To account for measurement error in the scales, correlations between variables were disattenuated using the formula


$$\begin{array}{l} r_{xy}^{*}=\;\frac{r_{xy}}{\sqrt{\alpha_{x}*\alpha_{y}}}, \end{array}$$

where *r*_*xy*_ is the observed Pearson correlation and α_*x*_ and α_*y*_ are the Cronbach's α values of the respective scales. This correction provides an estimate of the correlation as if the measures were perfectly reliable, allowing for a more accurate assessment of relationships between constructs. K-means clustering and hierarchical clustering were used to perform a classification of the sample into distinct psychological profiles. Free responses on open-ended questions were analyzed in a two-step lexical approach (see Section 2.2.3): (1) Translation of the individual responses into nominalized expressions and (2) categorization of the nominalizations based on content. Finally, the frequency of occurrence of the categories across the sample was counted for each question.

## 3 Results

### 3.1 Schema modes in music students

**Figure 1:**
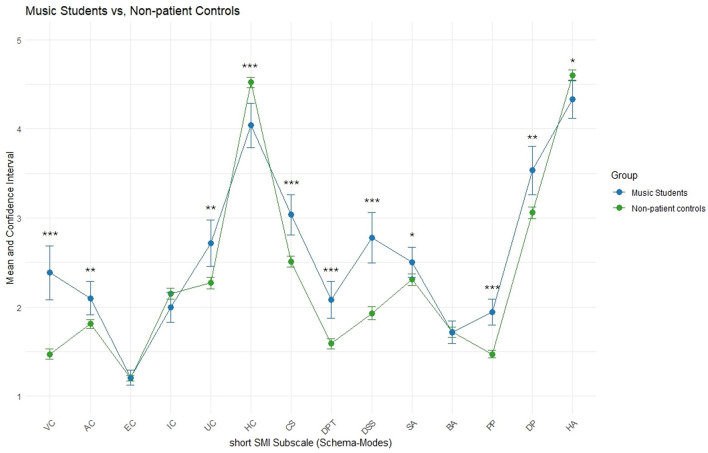
Music students compared to a non-clinical control group. Means and approximate 95% Confidence Intervals shown for short SMI subscales. Non-patient controls (*N* = 319) taken from available summary data (Lobbestael et al., 2010). Significance levels are based on approximate parametric procedures based on summary data and should be interpreted descriptively (*p < 0.05, **p < 0.01, ***p < 0.001).

**Figure 2:**
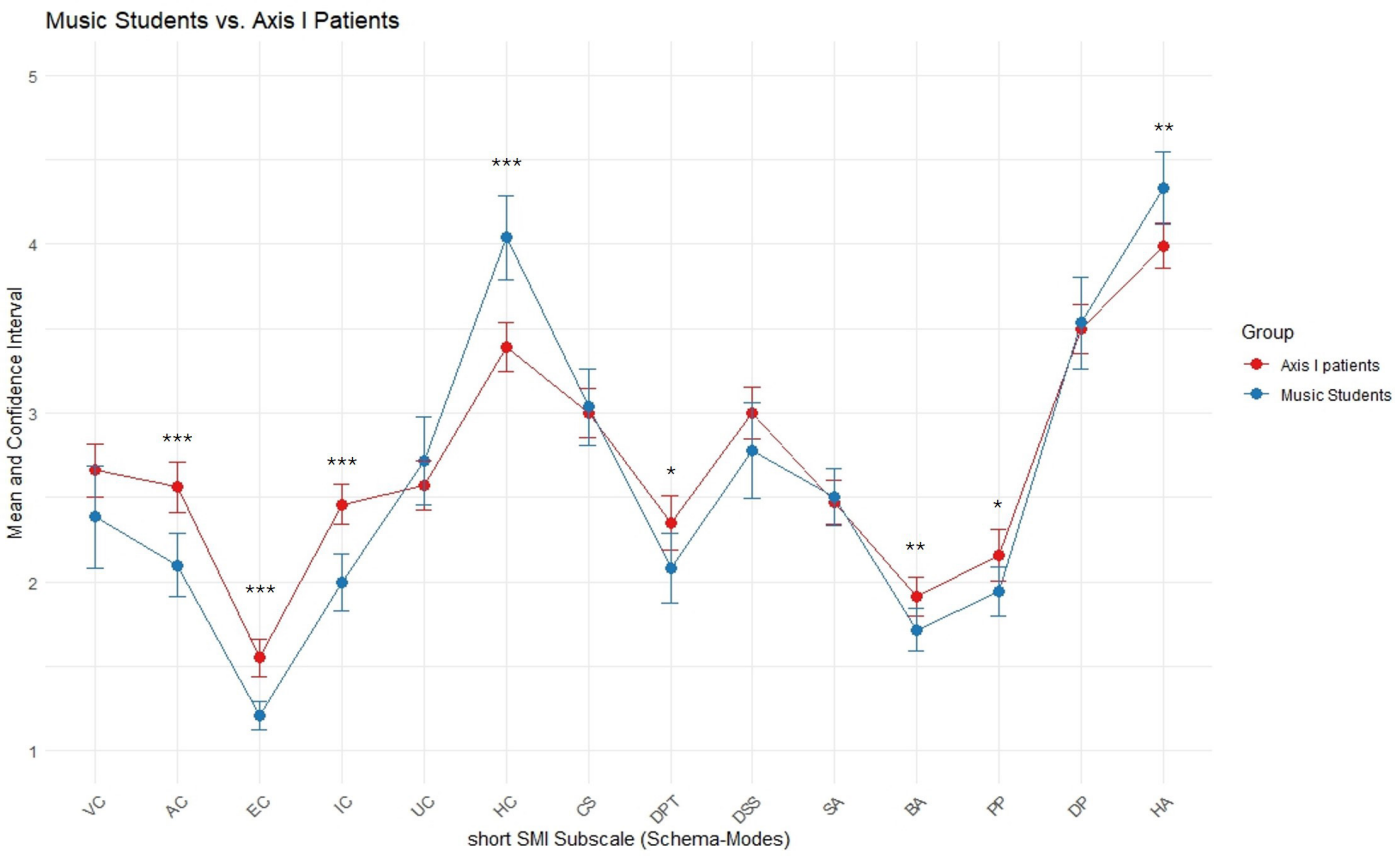
Music students compared to Axis I patients. Means and approximate 95% Confidence Intervals shown for short SMI subscales. Axis I patients (*N* = 136) taken from available summary data (Lobbestael et al., 2010). Significance levels are based on approximate parametric procedures based on summary data and should be interpreted descriptively (*p < 0.05, **p < 0.01, ***p < 0.001).

**Figure 3:**
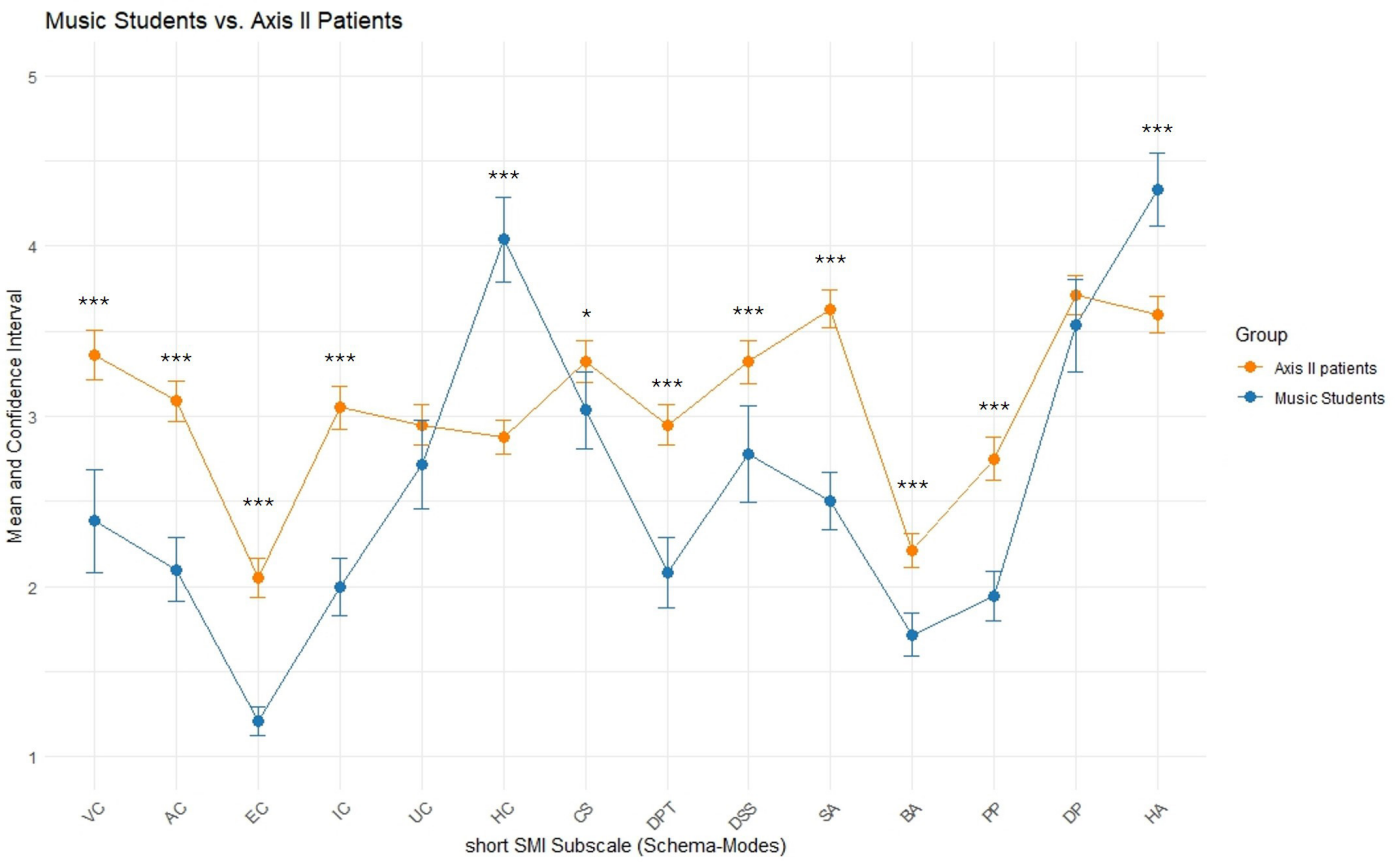
Music students compared to Axis II patients. Means and approximate 95% Confidence Intervals shown for short SMI subscales. Axis II patients (*N* = 236) taken from available summary data (Lobbestael et al., 2010). Significance levels are based on approximate parametric procedures based on summary data and should be interpreted descriptively (*p < 0.05, **p < 0.01, ***p < 0.001).

Scores on the short Schema Mode Inventory [short SMI (Lobbestael et al., 2010)] were compared with the three samples in the validation study of the questionnaire (Lobbestael et al., 2010): Non-patient controls, Axis I patients, and Axis II patients (see Table 2 for descriptive comparisons of effect sizes and Figures 1–3 for confidence intervals). These comparisons were based on the published summary data (means, standard deviations, and sample size) reported in the validation study (Lobbestael et al., 2010), as raw data were not available. As a consequence, all results in this section should be interpreted descriptively. Significance levels only indicate approximate parametric procedures based on available summary data. The validation study reports clinical groups classified according to Axis I (clinical disorders such as depression or anxiety) and Axis II (personality disorders) categories from the DSM-IV framework. While these classifications are now outdated in DSM-5/ICD-11, we used them here because the short SMI data provide large, clustered clinical samples (Lobbestael et al., 2010) that serve as a practical reference point for interpreting the relative severity and patterns of maladaptive and coping schema modes in our music student sample.

**Table 2:** Means, SD, and effect sizes of the short SMI sub scores for the music student sample compared to the summary data (means, SD) of the three samples of Lobbestael et al. (2010).

**Short SMI subscales**	**Music students (*****N*** = **46)**		**Non-patient controls (*****N*** = **319)**			**Axis I patients (*****N*** = **136)**			**Axis II patients (*****N*** = **236)**		
	**M**	**SD**	**M**	**SD**	δ**1**	**M**	**SD**	δ**2**	**M**	**SD**	δ**3**
VC	2.38	2.38	1.47	0.51	1.14^***^	2.66	0.94	−0.28	3.36	1.11	−1.24^***^
AC	2.10	0.63	1.81	0.48	0.51^**^	2.56	0.9	−0.59^***^	3.09	0.94	−1.24^***^
EC	1.21	0.29	1.2	0.29	0.03	1.55	0.67	−0.66^***^	2.05	0.92	−1.23^***^
IC	2.00	0.57	2.15	0.53	−0.28	2.46	0.72	−0.71^***^	3.05	0.97	−1.32^***^
UC	2.72	0.88	2.27	0.6	0.59^**^	2.57	0.85	0.17	2.95	0.94	−0.26
HC	4.04	0.84	4.52	0.54	−0.68^***^	3.39	0.87	0.76^***^	2.88	0.77	1.44^***^
CS	3.03	0.76	2.51	0.56	0.79^***^	3	0.88	0.04	3.32	0.95	−0.33^*^
DPT	2.08	0.69	1.59	0.52	0.81^***^	2.35	0.94	−0.32^*^	2.95	0.94	−1.05^***^
DSS	2.78	0.95	1.93	0.65	1.04^***^	3	0.91	−0.24	3.32	0.98	−0.56^***^
SA	2.50	0.57	2.31	0.59	0.33^*^	2.47	0.76	0.04	3.63	0.87	−1.54^***^
BA	1.72	0.42	1.72	0.51	−0.01	1.91	0.68	−0.34^**^	2.21	0.77	−0.80^***^
PP	1.94	0.50	1.47	0.39	1.06^***^	2.16	0.9	−0.30^*^	2.75	0.97	−1.04^***^
DP	3.53	0.91	3.06	0.6	0.61^**^	3.5	0.85	0.04	3.71	0.9	−0.20
HA	4.33	0.72	4.6	0.56	−0.41^*^	3.99	0.8	0.45^**^	3.6	0.83	0.94^***^

δ1 = Cohen's δ Music students vs. non-patient controls; δ2 = Cohen's δ Music students vs. Axis I patients; δ3 = Cohen's δ Music students vs. Axis II patients. Significance levels are based on approximate parametric procedures based on summary data and should be interpreted descriptively (^*^p < 0.05, ^**^p < 0.01, ^***^p < 0.001).

Cohen's d effect sizes were calculated relative to the published summary statistics of the short SMI validation study. While this allows descriptive comparisons between the music student sample and the normative and clinical groups, the absence of raw data limits the ability to conduct formal statistical tests or check distributional assumptions in the reference samples. In summary, the participants of the present study significantly differ descriptively in most of the short SMI scales from the non-patient controls in direction of the clinical Axis I and Axis II samples (samples from validation of short SMI, Lobbestael et al., 2010). At the same time, the sample showed considerable overlap with the scores of Axis I, but predominantly differed from Axis II scores.

Music students scored higher on all maladaptive modes except the Enraged Child (EC) and the Impulsive Child (IC) modes, as well as the Bully & Attack (BA) coping mode, than the non-clinical population (see Figure 1), reflecting worse coping with emotional triggers. Furthermore, significantly reduced adaptive modes (Healthy Adult, HA, and Happy Child, HC) reflect limited resources in adequate coping strategies. Considering Cohen's d effect size categorization (Sullivan and Feinn, 2012), Vulnerable Child (VC), Compliant Surrender (CS), Detached Protector (DPT) and Detached Self-Soother (DSS) Coping Modes as well as Punishing Inner Critic (PP) show large effect sizes; Angry Child (AC), Undisciplined Child (UC), Self-aggrandizer (SA) and Demanding Inner Critic (DP) yield medium effect sizes as do the reduced adaptive modes Happy Child (HC) and Healthy Adult (HA).

Compared to Axis I patients, music students reached similar, i.e., significantly non-different, scores on Vulnerable Child (VC), Undisciplined Child (UC), Compliant Surrender (CS), Detached Self Soother (DSS), Self-aggrandizer (SA), and Demanding Inner Critic (DP; see Figure 2), reflecting similar occurrence of these maladaptive emotional, cognitive and behavioral (coping) states as compared to these clinical patients. However, the present sample exhibited significantly lower scores (i.e., reduced occurrence of) on several other maladaptive modes than the Axis I patients: Angry Child (AC), Enraged Child (EC), Impulsive Child (IC), Detached Protector (DPT), Bully & Attack (BA), and Punishing Inner Critic (PP).

Compared to Axis II patients, music students reached similar, i.e., significantly non-different, scores only on Undisciplined Child (UC) and Demanding Inner Critic (DP; see Figure 3), reflecting a similar occurrence of these two maladaptive states as compared to these clinical patients. On all other maladaptive modes, the present sample exhibited significantly lower scores (i.e., reduced occurrence of) than the Axis II patients.

Moreover, music students exhibited significantly higher scores on adaptive modes (Healthy Adult, HA, and Happy Child, HC), showing that the participants in the sample have more availability of positive resources than Axis II patients—although less than the non-clinical control group (see above).

### 3.2 Internal consistency of the short SMI subscales

The internal consistency of the short SMI subscales in the present sample was generally good to excellent (following the guidelines of Nunnally and Bernstein, 1994). The Vulnerable Child (VC) mode showed very high reliability (α = 0.93, average *r* = 0.55), followed by Happy Child (HC, α = 0.89, *r* = 0.46) and Healthy Adult (HA, α = 0.86, *r* = 0.37). Other subscales showed good reliability: Detached Protector (DPT, α = 0.83, *r* = 0.36), Detached Self-Soother (DSS, α = 0.80, *r* = 0.50), Demanding Parent (DP, α = 0.82, *r* = 0.38), Angry Child (AC, α = 0.78, *r* = 0.25), Compliant Surrender (CS, α = 0.75, *r* = 0.30), Enraged Child (EC, α = 0.76, *r* = 0.32), Impulsive Child (IC, α = 0.72, *r* = 0.25), Undisciplined Child (UC, α = 0.72, *r* = 0.36), and Punishing Parent (PP, α = 0.70, *r* = 0.21).

The reliability analysis of the Bully and Attack subscale indicated low internal consistency (BA, α = 0.53, *r* = 0.14). The average inter-item correlation was *r* = 0.14, suggesting weak associations between items. Item-total correlations ranged from 0.10 to 0.40, with several items (e.g., Item 10, Item 19, and Item 28) contributing little to the overall scale. Several items displayed skewed response distributions and low item-total correlations, with most participants endorsing the lowest categories on several items (Item 24, Item 31, Item 20, and Item 14). This indicates pronounced floor effects with restricted variance and potential heterogeneity in the item content. Consequently, the scale provided limited variance and weak reliability in this sample. The reliability analysis of the Self-Aggrandizer subscale yielded a Cronbach's α = 0.64 (SA, α = 0.64, *r* = 0.15), which is at the lower threshold of acceptable internal consistency. Item-level inspection showed moderate corrected item–total correlations for most items, with two items (Item 12, Item 30) displaying weak associations, suggesting limited homogeneity within the scale. Deleting these items would slightly increase α, but the overall gain remained marginal. Thus, the subscale was retained in its original form for subsequent analyses.

No internal consistency was calculated for the short SMI full score, as it combines heterogeneous subscales measuring distinct maladaptive and adaptive schema-modes, and the small sample size relative to the large number of items would make the reliability estimate unstable.

Notably, the most reliable subscales (VC, HC, HA, DPT, and DSS) also showed the clearest and most consistent effects in subsequent analyses, including cluster analyses, correlations with other scales, and differences compared to the validation study samples. The two low-reliability subscales (SA and BA), reflecting strongly externalizing behaviors, yielded highly inconsistent responses (SA) and floor effects (BA), likely due to their low occurrence in the present sample and the socially undesirable nature of externalizing behaviors compared to internalizing behaviors (Robinson et al., 2019; Curhan et al., 2020). Taken together, these findings highlight that externalizing modes were less reliably captured in this sample of music students and may partly explain why these subscales rarely emerged as significant in subsequent analyses. Nevertheless, they were retained for theoretical completeness, while results involving these modes should be interpreted with caution. Overall, these reliability patterns provide additional confidence that the observed results for the majority of subscales reflect meaningful individual differences in schema-modes among the music students.

### 3.3 Interrelation between schema-modes and HIL scale

After reversing negatively poled items, participants in the sample on average scored low-medium (mean = 28.87, SD = 5.24) on the HIL scale. The HIL scale showed good internal consistency (Cronbach's α = 0.79) and an average inter-item correlation of 0.36 (see Supplementary Table 1). Although no general norms exist for the HIL scale, previous studies with music students and teachers reported mean scores between approximately 30 and 33, which can be considered as reflecting moderate to good coping (Hildebrandt et al., 2012). By contrast, pre-intervention values in music teachers (M = 26.8) and advanced students (M = 24.6) were lower, with improvements to around 29–30 after intervention (Hildebrandt, 2004). In this context, the present sample's mean of 28.9 can be interpreted as slightly lower but still within an average to moderate–good range of coping and above the pre-intervention levels reported for teachers and advanced students. There was no difference between the HIL scores of female (mean = 29.07, SD = 5.05) and male (mean = 29.79, SD = 4.66) musicians [*t*_(27.77)_ = 0.46, *p* = 0.649 n.s.], with not enough data available for participants who selected gender “other/prefer not to say.”

**Figure 4:**
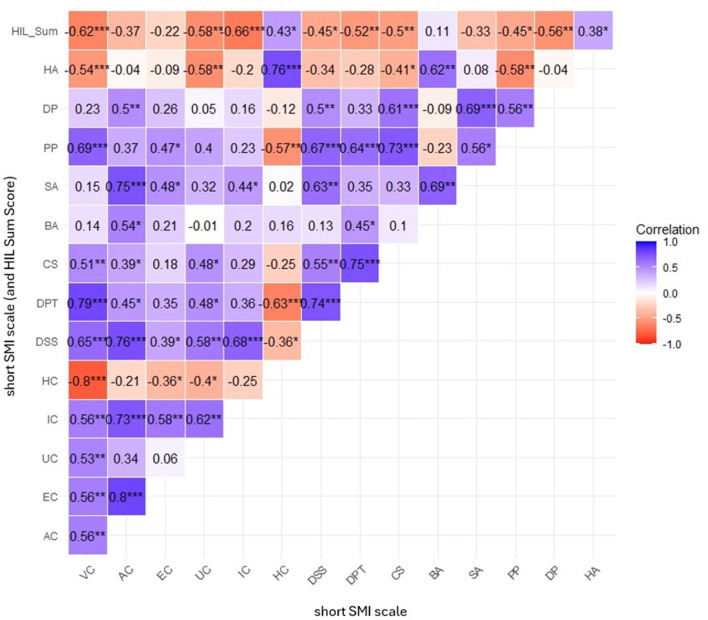
Disattenuated correlation matrix of SMI schema-modes and HIL total score. Pearson correlations corrected for scale reliability. Significance levels: *p < 0.05, **p < 0.01, ***p < 0.001.

All maladaptive schema modes of short SMI were negatively correlated with HIL sum score (see Table 3 for observed and disattenuated correlations): The higher the score on the respective maladaptive modes (see Figure 4 for Correlation Matrix with disattenuated correlations**)**, the lower the scores on the HIL sum score, indicating worse capabilities to cope with life as a musician. Solely modes related to rage and anger [EC (enraged child), AC (angry child), and BA (Bully & Attack)] did not yield significance with respect to correlation with the HIL sum score. In contrast, high expression on Happy Child (HC) and Healthy Adult (HA) was positively correlated with HIL sum score (*r* = 0.36, *r*_*disattenuated*_ = 0.43, *p* < 0.015, respectively, *r* = 0.31, *r*_*disattenuated*_ = 0.38, *p* < 0.038). Consequently, higher scores on these positive, adaptive schema-modes were associated with better coping with life as a musician in the sample.

**Table 3:** Observed (top row) and disattenuated (bottom row) correlations between HIL sum score and SMI modes.

**Statistical metric**	**HIL Score**	**VC**	**AC**	**EC**	**UC**	**IC**	**HC**	**DSS**	**DPT**	**CS**	**BA**	**SA**	**PP**	**DP**	**HA**
Observed Pearson correlation	HIL sum score	−0.53^***^	−0.29	−0.17	−0.44^**^	−0.5^***^	0.36^*^	−0.36^*^	−0.42^**^	−0.39^**^	0.07	−0.24	−0.34^*^	−0.45^**^	0.31^*^
Disattenuated correlation (corrected with Cronbach's alpha)	HIL sum score	−0.62^***^	−0.37	−0.22	−0.58^**^	−0.66^***^	0.43^*^	−0.45^*^	−0.52^**^	−0.5^**^	0.11	−0.33	−0.45^*^	−0.56^**^	0.38^*^

Significance levels: ^*^p < 0.05, ^**^p < 0.01, ^***^p < 0.001.

Interrelations between the schema-modes reflect predictions by schema theory. Emotional triggers usually lead to activation of emotional child modes, cognitive parent modes (inner critic), and a maladaptive behavioral coping response. Only in the case of sufficient resources of the “Healthy Adult” (HA), activation of maladaptive coping responses is reduced, and the intensity of emotions and cognitions by child and parent modes can be attenuated. It is especially noteworthy that a high negative correlation between “Happy Child” (HC) and “Vulnerable Child” (VC) modes exists (*r* = 0.73, *r*_*disattenuated*_ = 0.80, *p* < 0.001) next to smaller negative correlations between HC and other child modes (see Figure 4 for correlation matrix with disattenuated correlations). Furthermore, the VC mode shows strong correlations with all other maladaptive child modes (AC, EC, IC, and UC). It is also closely linked to “freeze” and “flight” coping responses (CS Compliant Surrender, DPT Detached Protector, and DSS Detached Self-Soother). In addition, the VC mode is associated with punishing self-talk (PP Punishing Parent Mode), which often manifests as intense negative cognitions such as self-hate and vulnerable feelings of inferiority, shame, or worthlessness. This is also indicated by the high correlation between PP and VC in comparison to the other child modes with externally directed emotions (anger and impulsiveness). Conversely, those outwardly directed child emotions of the AC (Angry Child) are exclusively correlated with the Demanding Parent mode (DP). Moreover, high scores on maladaptive child and parent modes—reflecting frequent occurrence of these stages in the participants—are related to increased occurrence of maladaptive coping responses of at least one category, with the “freeze” and “flight” states being the most frequently experienced states.

### 3.4 Clustering analysis: 3 distinct psychological profiles of music students

To investigate whether specific psychological and coping profiles of music students exist, we performed an exploratory cluster analysis on the short SMI sub-scores. The Within-Sum-of-Squares (WSS) plot showed a monotonic, exponential decrease of WSS for increasing cluster size, with a visual estimation of an “elbow” at around three clusters. The three-cluster solution was confirmed by the dendrogram of the exploratory hierarchical cluster analysis and is an adequate number of clusters for a sample size of *N* = 46.

**Figure 5:**
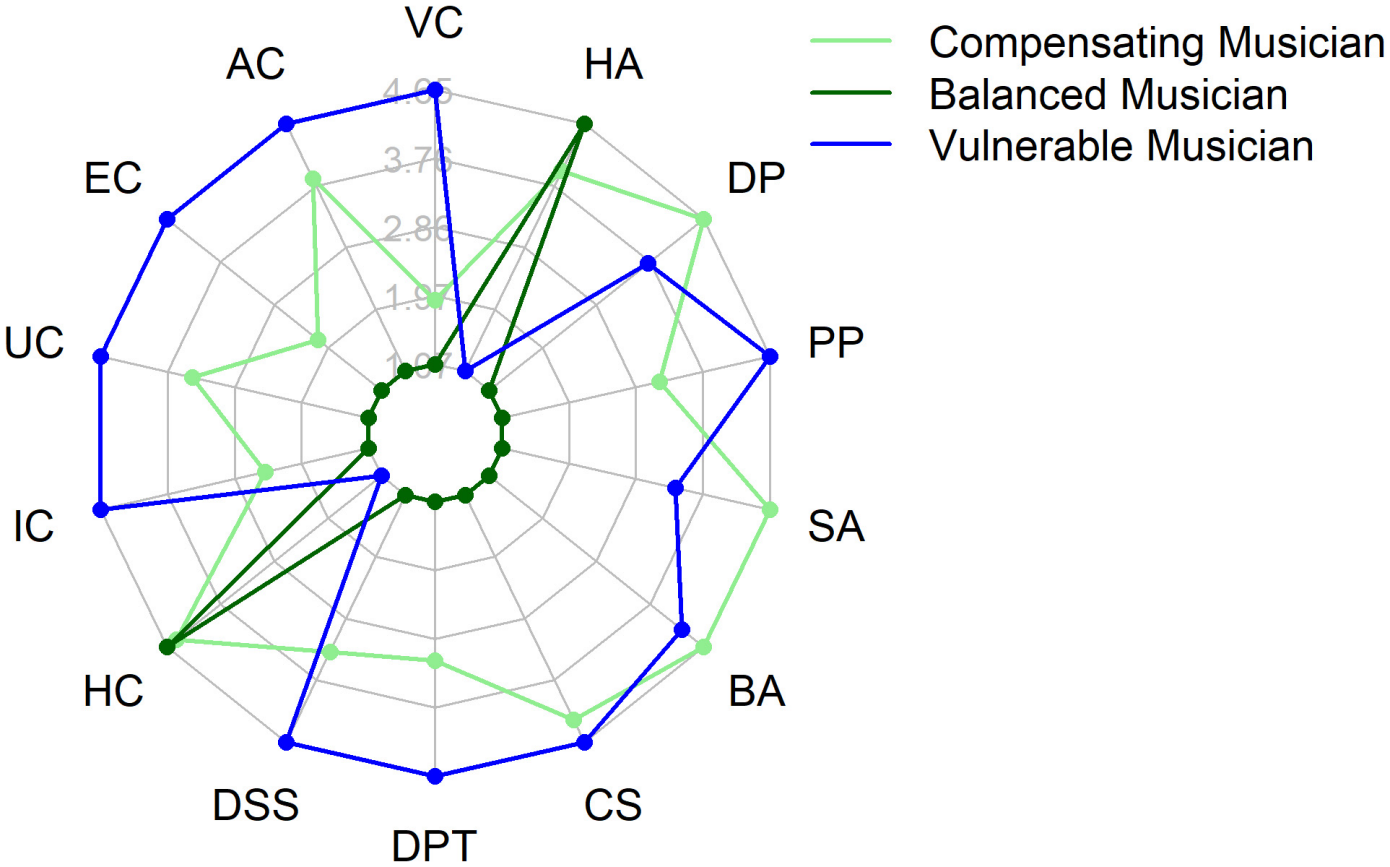
Psychological Profiles based on SMI modes of the three clusters of music students. light green = Cluster 1 (“Compensating Musician”), green = Cluster 2 (“Balanced Musician”), blue = Cluster 3 (“Vulnerable Musician”). To scale the graphic for better visual interpretation and comparability of the values, two additional lines were included in the plot: an upper limit (maximum value) and a lower limit (minimum value) for each variable. These boundary values represent the maximum and minimum values of the individual variables across all clusters, ensuring that the values are presented within the same scale. As a result, the group means are depicted within this scale, allowing for a clearer visualization of the relative differences between the variables and groups. The maximum and minimum values of each axis serve solely as a reference framework and are not identical to the actual means of the variables within the respective groups. They provide a visual unification of the scale, making it easier to observe the relative differences between the variables. This means that the axes themselves do not directly reflect the means but rather allow for a visual standardization of the variables, emphasizing comparative differences between the groups.

The three obtained clusters significantly differ on HIL sum scores [*F*_(42, 2)_ = 8.216, *p* < 0.000974, η^2^ = 0.281] with Cluster 1 (*N* = 21, Δ mean = −7.21, CI [−2.638, −11.79], *p* < 0.010) and Cluster 3 (*N* = 10, Δ mean = −4.81, CI [−0.996, −8.623], *p* < 0.001) showing lower overall capability to cope with the life as a musician compared to Cluster 2 (*N* = 14; compare Table 4). The distinguishing correlation between cluster number and HIL sum score, as well as the characteristic profile of the presence and intensity of maladaptive, respectively, adaptive (HC and HA) short SMI subscores (schema modes), indicates a meaningful cluster analysis (see Table 4). The radar plot profiles displayed in Figure 5 for each group are based on the means of the respective variables for each cluster level, scaled relatively between SMI modes and clusters.

**Table 4:** Means (and SD) for HIL sum score and SMI modes characterizing the clusters of music students: cluster 1 (“Compensating Musician”), cluster 2 (“Balanced Musician”), and cluster 3 (“Vulnerable Musician”).

**Cluster**	**Size**	**HIL (sum)**	**VC**	**AC**	**EC**	**UC**	**IC**	**HC**	**DSS**	**DPT**	**CS**	**BA**	**SA**	**PP**	**DP**	**HA**
			**Maladaptive child modes**					**Happy child**	**Maladaptive coping modes**					**Maladaptive inner self-talk**		**Healthy adult**
1	*N* = 21	27.90 (4.76)	2.17 (0.52)	2.27 (0.41)	1.19 (0.19)	2.88 (0.94)	1.98 (0.46)	4.32 (0.56)	2.99 (0.67)	2.19 (0.51)	3.31 (0.50)	1.79 (0.45)	2.82 (0.45)	2.01 (0.499)	3.95 (0.82)	4.46 (0.60)
2	*N* = 14	32.71 (3.47)	1.64 (0.34)	1.57 (0.32)	1.07 (0.15)	2.06 (0.50)	1.62 (0.37)	4.39 (0.55)	1.84 (0.55)	1.48 (0.45)	2.37 (0.70)	1.57 (0.45)	1.99 (0.40)	1.55 (0.26)	2.82 (0.85)	4.65 (0.49)
3	*N* = 10	25.50 (5.36)	3.89 (0.84)	2.47 (0.88)	1.46 (0.45)	3.30 (0.55)	2.55 (0.61)	2.96 (0.81)	3.65 (0.79)	2.71 (0.67)	3.40 (0.72)	1.77 (0.31)	2.53 (0.53)	2.34 (0.41)	3.66 (0.54)	3.63 (0.81)

### 3.5 Open questions: inner self-talk and complaints related to making music

Free responses on open-ended questions were analyzed in a two-step lexical approach: (1) Translation of the individual responses into nominalized expressions and (2) categorization of these expressions into descriptive categories. Finally, the frequency of the occurrence of the categories across the sample was counted for each question (see Table 5 and Supplementary Table 2 for coding scheme and example quotes).

**Table 5:** Descriptive categories of free responses (multiple responses possible).

	**If you have complaints related to making music, what are they? (Question 1)**	**If you have/have had problems with a teacher or orchestra or chamber music partner currently or in the past, what problems were they? (Question2)**	**What are typical thoughts (positive and negative) you have in musical situations (practice, rehearsal, lesson, stage)? (Question 3)**	
Categorized free responses (multiple responses possible)	(*N* = 18)	(*N* = 19)	Positive (*N* = 16)	Negative (*N* = 21)
	Pain (6)	Communication (10)	Joy/pleasure (5)	Self-doubt (10)
	Self-criticism/own expectations/comparison with others (5)	Lack of quality of teaching (pedagogical method, technique, inadequate feedback) (8)	Goal-orientation (4)	Feeling of inferiority/insufficiency (9)
	Injuries (e.g., tendonitis, inflammation, etc.) (3)	Social interaction & organization in ensemble (5)	Realistic self-reflection/self-structuring (4)	Opinion of/comparison with others (8)
	Tension/physical discomfort (3)	Distress due to the mood of the teacher (4)	Connection with others/belonging (4)	Demanding self-criticism (5)
	Confidence on stage/stage fright (2)	Lack of respect/sexism/discrimination (3)	Gratefulness (3)	Fear/nervousness (5)
	Work-life-balance/self-organization (2)	Anxiety/stress to perform (1)	Flow (3)	Hyperfocus on body or technique/errors (5)
	Expectations of others (2)		Process-orientation (3)	Shame/not belonging (4)
	Dysphonia (2)		Curiosity/surprise/excitement (3)	Punishing self-critic/self-hate (4)
	Social conflicts (1)		Confidence/pride (2)	Frustration/annoyance (3)
			Imagination (1)	Hopelessness/disappointment (3)
				Fear of future/not good enough (3)

Frequencies reflect the number of participants mentioning each category at least once per question. Multiple responses from a single participant across different categories were counted, while repeated responses within the same category were counted only once per participant per question.

Apart from physical (e.g., pain-related) issues, most frequently mentioned complaints (Question 1) relate to destructive self-talk and expectations of self and others, as well as topics of social interaction and organization. The latter aspect is strongly reflected in the responses to Question 2: “If you have/have had problems with a teacher or orchestra or chamber music partner currently or in the past, what problems were they?” Most frequent categories relate to social communication and interaction issues within music ensembles and between the music teacher and the music student.

During practice, rehearsal, and stage situations, the most frequently reported negative thoughts and feelings fell into four descriptive categories: (1) self-doubt, (2) feeling of inferiority/insufficiency, (3) externally oriented worrying (comparison/opinion of others), and (4) self-critical thoughts of demanding, punishing, and hyperfocusing type. Nevertheless, several participants mentioned joy/pleasure, goal and process orientation, connection with others through music, and flow states as positive resources and thoughts and feelings on stage.

Responses on open-ended questions did not differ in content between the three clusters, though the small sample size in each cluster and the design of the questions as voluntary limits the interpretability and generalizability of this comparison. The summary mentioned above thus provides a descriptive overview of typical issues, complaints, and inner self-talk among music students (Dews and Williams, 1989), rather than a quantified comparison of positive vs. negative thoughts or relative frequencies of complaints.

## 4 Discussion

The application of the short Schema Mode Inventory [short SMI (Lobbestael et al., 2010)] in this study represents a novel approach in psychological research on music students, providing valuable insights into their schema modes and their psychological profiles based on inferential analyses within our own sample and comparisons to the available summary data of non-clinical controls and Axis I and II patients from representative samples reported in the validation study (Lobbestael et al., 2010). Our findings suggest that music students exhibit scores on maladaptive child modes (VC, AC, and UC) and coping modes substantially elevated compared to non-patient controls, aligning more closely with Axis I patients and, in part, overlapping with Axis II patients for maladaptive modes. This is consistent with the monotonic increase in symptom severity with higher maladaptive short SMI scores reported in the validation study (Lobbestael et al., 2010) and suggests that music students, while not presenting clinical disorders *per se*, experience emotional and psychological challenges intermediate between non-clinical controls and clinical populations.

Particularly notable were the large descriptive effect sizes for Vulnerable Child (VC), Compliant Surrender (CS), Detached Protector (DPT), Detached Self- (DSS), and Punishing Parent (PP), indicating substantial psychological distress compared to non-clinical controls (see Table 1 for mode descriptions). With the exception of PP, these modes—along with the demanding parent (DP) mode—showed no significant distinction from Axis I patients. These findings are compatible with subclinical or undiagnosed Axis I disorders such as depression or anxiety disorders, as patients with anxiety disorders and depression have been shown to have heightened early maladaptive schemas (Hawke and Provencher, 2011). However, the presence or absence of Axis I disorders in our sample cannot be determined from the present study. Interestingly, in the short SMI validation study (Lobbestael et al., 2010) VC scores were strongly correlated with loneliness (Loneliness Scale, LS, *r* = 0.71), fearful attachment style in relationships (RSQ fearful attachment, *r* = 0.77), and childhood trauma (Childhood Trauma Questionnaire, CTQ, *r* = *0.7*9), as hypothesized *a priori* by the authors of the short SMI (Lobbestael et al., 2010). The high scores on CS, DPT, DSS, and PP in the present study furthermore correlated at medium to high levels with the very same scales in the validation study (Lobbestael et al., 2010), although this was unexpected by the authors. Although trauma history was not assessed in the present study, these correlations of short SMI (Lobbestael et al., 2010) raise the hypothesis that adverse childhood events or traumatic experiences, insecure attachment, and loneliness contribute to the particularly increased maladaptive modes in music students. However, this cannot be inferred directly from the current study and warrants future investigation.

Following this hypothesis and based on theoretical considerations, the present results in music students appear consistent with previous research indicating loneliness and social isolation in musicians (Korte et al., 2025; Kapsetaki and Easmon, 2019; Iorwerth and Knox, 2019; Cardoso et al., 2025) as well as traumatic childhood experiences as a risk factor for musicians' dystonia (Schneider et al., 2021; Alpheis et al., 2022, 2023). Heightened early maladaptive schemas and schema modes have been found in patients with psychological trauma and PTSD (Karatzias et al., 2016; Price, 2007). Interestingly, Rezaei et al. (2016) found that especially the schema “rejection” mediated the adverse effect of childhood trauma on the development of depression, Baník et al. (2022) found the schemas “defectiveness” and “failure” linked to depression, and Dutra et al. (2008) found increased suicidality in trauma patients with heightened scores on both “defectiveness,” “failure,” and “social isolation” schemas. Given that experiences of rejection and feeling inferior or defective compared to other musicians are reported in this study (see section “Inner Self Talk” below), and are common in the highly competitive performance industry of professional musicians (Skaggs, 2019; Swart, 2016; Nagel, 1990; Raeburn, 2000), e.g., in competitions or engagements for concerts and collaborations with artist managers and recording labels, stress and pressure from rejection or perceived inferiority would be expected to trigger early maladaptive schemas in musicians with trauma history or previous adverse experiences with harsh or unconstructive criticism. Such processes would be expected to contribute to heightened emotional reactions (child modes) and more maladaptive coping strategies (“fight-flight-freeze” responses) such as perfectionism, procrastination, self-soothing with addictive behavior, sublimation, or narcissistic attitudes, which in turn could affect wellbeing or career development. However, these interpretations—though theoretically grounded and consistent with existing literature—remain speculative and require direct empirical investigation in musicians with documented trauma histories.

Reduced scores in adaptive modes [Healthy Adult (HA) and Happy Child (HC)] compared to non-clinical controls—although significantly higher than in both clinical populations—suggest limitations in positive coping resources and indicate that supportive interventions would be beneficial. Research on schema-focused interventions is needed to confirm this hypothesis. While there is only non-significant overlap with Axis II patients (personality disorders) on UC (Undisciplined Child) and DP (Demanding Parent) in the validation study (Lobbestael et al., 2010), the results have to be interpreted carefully, since people with personality accentuations, e.g., narcissistic tendencies (Dora, 2025) typically do not voluntarily participate in psychological studies or offerings because of their belief that others are the problem.

The HIL Scale results revealed a lower average level of coping among the music students, with no significant gender differences. This contrasts with previous findings (Hildebrandt et al., 2012) in which researchers investigated psychological distress in a longitudinal study of 105 first-year students at three Swiss music universities and found a greater increase in the tendency to exhaustion among women compared to men in the first year of their studies. Whether these differences can be explained by a greater willingness to deny complaints in men remains unclear (Sokoli et al., 2022). In the present study, the mean HIL score of 28.9 was somewhat lower than the 31–33 typically reported for first-year students in earlier research (Hildebrandt et al., 2012), but at the same time, clearly higher than the pre-intervention values documented for teachers and advanced students (Hildebrandt, 2004), which likely reflected groups with above-average psychological strain. This suggests that our sample demonstrated slightly reduced coping resources compared with typical student cohorts, but not to the extent observed in pre-intervention groups with elevated distress.

Maladaptive schema modes were negatively correlated with HIL scores, underscoring the impact of psychological profiles on musician-specific coping capabilities. These correlations suggest that musicians who frequently operate from maladaptive patterns (e.g., feeling overwhelmed, self-critical, or detached) show impaired ability to cope with music-specific demands, including confidence in stage situations, satisfaction with performance, and physical self-regulation (e.g., posture, breathing, movement control). This is particularly noteworthy for musicians, as their profession often involves high-pressure performance situations where effective coping is crucial for sustained wellbeing and career longevity. Positive correlations between the HIL Scale and HC and HA modes indicate that the presence of adaptive modes enhances music-specific coping, reflecting Schema Theory's predictions of reduced maladaptive coping responses with stronger Healthy Adult resources. Specifically, these results imply that musicians who can access their Healthy Child (HC) mode for joy and spontaneity, and their Healthy Adult (HA) mode for emotional regulation, self-reflection, thoughtful decision-making, and self-nurturing, are better equipped to manage the unique stressors of their musical lives. While the general principle that adaptive psychological resources support coping aligns with broader Schema Theory (as evidenced by the reported reverse relationship between HA and HC and the severity of Axis I and Axis II symptoms in general populations (Lobbestael et al., 2010) in the validation of short SMI), our study specifically extends this understanding to the domain of music performance. The purpose of presenting these correlations is to demonstrate how established schema theory constructs manifest within the distinct context of music students, highlighting the practical relevance of schema modes for understanding and potentially improving their coping mechanisms in a performance-oriented field.

The interrelation among maladaptive scores underscore the dynamic interplay within the schema mode system, where emotional triggers frequently activate maladaptive Child modes, inner critics (Parent modes), and subsequent maladaptive coping responses. This observed interrelation between several Child modes (Emotions), Parent modes (Cognition and internalized Beliefs), and coping behaviors is typical for schema activation, as described in the introduction. Crucially, the presence of a robust Healthy Adult mode appears to mitigate these maladaptive reactions, allowing for a reduction in the intensity of distressing emotions and cognitions, while the Vulnerable Child mode emerges as a central driver of psychological distress and dysfunctional coping.

The patterns of internal consistency observed in the Short-SMI subscales provide meaningful context for interpreting the results. Subscales with high reliability, such as Vulnerable Child (VC), Detached Protector (DPT), Detached Self-Soother (DSS), Happy Child (HC), and Healthy Adult (HA), were not only measured consistently but also corresponded to the modes showing the strongest effects across analyses, including cluster membership (see Section 4.1) and correlations with coping (HIL) scores. This suggests that these consistently measured modes are central to characterizing emotional vulnerability, coping strategies, and psychological resources in music students.

In contrast, the two subscales Self-Aggrandizer (SA) and Bully & Attack (BA) showed low internal consistency. Item-level diagnostics revealed several items with very low corrected item–total correlations, and the removal of individual items produced only marginal changes in Cronbach's α, indicating that the low reliability likely reflects genuine measurement limitations in this sample rather than the influence of a single problematic item. Both scales also displayed low mean endorsement and restricted variability, with positively skewed and leptokurtic distributions, suggesting that these socially undesirable behaviors occurred infrequently and were endorsed by only a few participants. Such low base-rate responding and potential social desirability effects may have contributed to inconsistent response patterns. Taken together, these findings highlight that the most reliably measured and frequently expressed modes are the most informative for understanding the psychological profiles of music students, whereas less frequently expressed, externalizing modes such as SA and BA should be interpreted with caution.

### 4.1 Clustering profiles: identification of psychological subtypes

To investigate whether specific psychological profiles exist among music students, we performed an exploratory cluster analysis on the short SMI sub-scores. The analysis revealed three distinct profiles, which we preliminarily described as: “Balanced Musician” (*N* = 14), “Compensating Musician” (*N* = 10), and “Vulnerable Musician” (*N* = 21; compare Figure 5). Given the exploratory nature of this analysis and limited sample size (*N* = 46), the following cluster descriptions should be considered preliminary hypotheses requiring validation in larger, independent samples. Hence, the following descriptions and interpretations are based on the analysis of the schema modes in each cluster and theoretical assumptions from schema theory and schema-focused methods (Young et al., 2006; Wenhart, 2024; McCormick et al., 2022; Migge, 2014).

The “Balanced Musician” cluster showed the highest overall coping capability, characterized by lower scores on maladaptive modes and higher scores on adaptive modes. These musicians appear to maintain stable emotional states and employ effective coping strategies.

The “Vulnerable Musician” cluster demonstrated the most significant psychological distress, marked by high scores in maladaptive child and passive coping modes (“Freeze” and “Flight”) and low scores in adaptive modes. These individuals likely experience frequent, intense negative emotions and attempt to avoid them through, e.g., fleeing the stressor or pleasing others (interpersonally or by musical perfectionism). These students would likely benefit from comprehensive individual support to address their emotional vulnerabilities effectively. Further research is needed to determine the extent and form of such interventions.

The “Compensating Musician” cluster exhibited medium levels of maladaptive modes and slightly lower scores on adaptive modes, reflecting a group that seems to employ compensatory strategies to manage their emotional and psychological challenges superficially. These musicians may rely more often on externalizing coping behavior (“Fight,” e.g., self-aggrandizing, “bully & attack”) and validation by others (“Freeze,” e.g., CS, compliant surrender) to navigate their stressors and perform in the short term. Although they seem to have more healthy resources (HA, HC) than “vulnerable musicians,” they may be at risk of decompensating if stressors increase, resources diminish, or when success fails to materialize. Compared with “vulnerable musicians,” “compensating musicians” report fewer emotional child modes. This pattern could reflect either limited emotional awareness or social desirability bias in their reporting.

Hypothesizing about a potential relation to performance-related stress and anxiety, these cluster profiles show conceptual similarities to Spahn and Krampe's MPA typology (Spahn et al., 2021b), suggesting that “Balanced Musicians” show similarity to the Type I MPA group (presumably low MPA, common Lampenfieber, Spahn et al., 2021b) with high self-efficacy and adaptive coping, while “Compensating Musicians” potentially show increased performance stress but may still have strategies to cope with it and regulate themselves during performance (presumably high MPA at the beginning of the performance, Spahn et al., 2021b). “Vulnerable Musicians” may be at risk of getting into a vicious circle on stage by insufficient coping strategies and emotional regulation (presumably Type II MPA, which worsens during performance, Spahn et al., 2021b). Research is needed to investigate the relationship between MPA severity and type and schema-mode expression.

### 4.2 Qualitative insights: inner self-talk and complaints related to making music

The analysis of free-response questions revealed common themes related to physical pain, self-criticism, social interaction, and organizational issues. Negative self-talk and self-doubts were prevalent, especially during musical tasks, indicating the need for interventions addressing destructive inner dialogues. These reports reflect the heightened demanding and punishing parent modes (DP and PP) in the study sample. A considerable number of subjects mentioned difficulties in social communication and interaction with teachers, conductors, and colleagues as distressing factors. Social relationships are among of the most important factors in predicting wellbeing and healthy aging in the general population (Baldassare et al., 1984; Reis and Gable, 2003; Clarke and Nieuwenhuijsen, 2009). This is especially noteworthy as Ascenso et al. (2017) identified the “shared nature of music making” and the “oneness in performance with others” as crucial for the experience of meaning and purpose in professional musicians. Moreover, emotional distressing student–teacher relationships are frequently related to mental health issues in the further course of the musical career and traumatic familial or pedagogical experiences may contribute to the risk of musicians' dystonia (Schneider et al., 2021). While these reports are subjective and cannot be independently verified, students' descriptions of disrespect or pedagogical qualification of teachers, as well as sexism and discrimination in teacher–student relations and music ensembles, reflect perceived emotional abuse and distress.

Sexual and emotional abuse among students is a recognized problem across educational contexts and is associated with long-term negative effects on wellbeing and development (Steele et al., 2024; Boskovic et al., 2023; Özevin, 2022; Ramstedt, 2023; Spadine et al., 2022; Elpus and Carter, 2016). In music education, evidence shows that teacher-perpetrated abuse is common, with more than one-quarter of students reporting emotional abuse and up to 10% physical abuse in school music classes (Özevin, 2022). Reported physical acts by music teachers included beating, pulling ears or hair, hitting a student's head with instruments or rulers, and throwing objects, while emotional abuse ranged from harsh criticism, insults, and neglect to humiliating practices such as comparing a student's voice to an animal sound, making fun of physical characteristics, or forcing peers to spit on a singled-out child (Özevin, 2022). In higher education, qualitative research highlights similarly patterned emotional abuse embedded in classical music culture, including humiliation, harmful comparison, and verbal aggression (Ramstedt, 2023). In contrast, peer-perpetrated bullying disproportionately affects music and theater students, with male students being more vulnerable to physical aggression and female students to social/relational victimization (Elpus and Carter, 2016).

Positive responses of the participants highlighted resources such as joy, goal orientation, connection through music, and flow experiences. Such positive emotions are crucial for general wellbeing (Ascenso et al., 2017; Fredrickson, 2003) and effective coping in dealing with performance anxiety (Spahn et al., 2021a). Especially, positive feelings can increase and stabilize internal resources and resilience by means of the broaden and build effect (Fredrickson, 2001): positive emotions increase wellbeing and positive action repertoire, which in turn further increase wellbeing and resources. Furthermore, flow experiences (Csikszentmihalyi and Csikzentmihaly, 1990) and feelings of accomplishment and process orientation (Deci and Ryan, 2012) have been associated with increased motivation and creativity (Csikszentmihalyi and LeFevre, 1989; Ryan and Deci, 2008; Ryan et al., 2008; Deci and Ryan, 2008).

### 4.3 Implications for prevention and interventions at music school

These distinct psychological profiles suggest that music students would benefit from personalized coaching interventions. Due to the exploratory nature of the cluster analysis and the small sample size, further research is needed to validate the existence of the three coping clusters described for the first time in the present study. As a second step, psychological interventions targeting hypothetically different needs of the coping clusters could be developed and investigated. The following implications and recommendations for prevention and interventions at music schools are based on the schema-mode presence in the three preliminary clusters of the present study and theoretical considerations of existing literature on schema-focused coaching methods (Young et al., 2006; Wenhart, 2024; Migge, 2014).

“Balanced Musicians” would likely benefit from programs aimed at maintaining and refining their existing coping strategies. Coaches can incorporate advanced techniques for stress management, performance optimization, and emotional regulation. Encouraging practices that emphasize balance and wellbeing, such as regular physical exercise, structured practice routines, and social support, can help these individuals sustain their adaptive coping mechanisms. Such interventions are already implemented by a wide range of music universities via the course programs in the field of music physiology and musicians' medicine (e.g., stage training, mental training, or body-focused programs such as Alexander Technique or Dispokinesis). Whether the increasing efforts at conservatoires with regard to prevention and therapy will be able to compensate for the frequent musician-specific complaints or only alleviate them remains to be seen at the present time. Initial empirical values have been documented, at least for conservatoire training (Spahn et al., 2016; Hildebrandt, 2009; Steyn et al., 2016).

For “Compensating Musicians,” strategies should focus on building internal resilience and self-validation. This group may benefit from techniques of schema-focused coaching that strengthen adaptive modes (HA and HC) and enhance internal coping mechanisms, such as mindfulness practices, resilience training, and schema-focused group workshops as suggested by Wenhart Wenhart (2024). Similarly, “Vulnerable Musicians” benefit from such techniques, but some may need comprehensive support in terms of individual psychological coaching or even long-term psychotherapy. Dedicated training for both groups should focus on dealing with negative inner self-talk on stage, but equally during practice and in ensemble playing. Imaginative techniques, role plays on chairs between schema modes, and exercises to increase self-confidence should be central to workshops and coaching to increase self-efficacy and adaptive coping as suggested by Wenhart (2024) and in accordance with the broaden-and-build effect (Fredrickson, 2001) and the self-determination theory (Ryan and Deci, 2008; Ryan et al., 2008). Additionally, psychoeducation on the interaction between biological predisposition (e.g., stress sensibility and nervous system activity), present and past social system, and psychological factors (inner self-talk, beliefs, etc.) may be taught to enable music students to individually reflect and work on their unique levers. However, these recommendations are preliminary and should be tested empirically before implementation.

A comparable voluntary workshop, as suggested by Wenhart (2024) was conducted for the participants of the present study as a pilot workshop, which yielded overall positive feedback. More than twice as many participants enrolled in personal 1:1 coaching and greatly appreciated the offer, underlining the need for a confidential space to target individual topics with a psychological expert. Such coaching with psychologically educated personnel could represent a promising approach to help musicians deal with individually experienced traumatic or non-traumatic emotional issues in their past personal or musical life, as intense traumatic or recurring similar emotional situations create schemas that are automatically triggered on stage or in musical interaction with others, as soon as the brain detects a similar emotional threat.

Finally, collaborations with external psychological or psychiatric clinics and implementing peer-support groups could help to facilitate access to mental health support and reduce stigma.

### 4.4 Limitations and future research

A limitation inherent to the online study design is the inability to control for the representativeness of the sample. While we made sure to distribute the questionnaire widely across the participating Swiss music universities, participation likely depended on students' personal interest in and attitudes toward psychological topics. Participants may have had heightened interest in psychological topics or concerns about their mental health, whereas non-participants may have perceived stigma. Additionally, comparisons with the published validation study samples were based solely on summary data, as raw data were not available, and should be interpreted descriptively. Despite these constraints, the analyses conducted within our dataset were inferential and yielded consistent and striking results. The limitations primarily concern the external comparisons and the degree to which findings can be generalized, rather than the validity of the statistical methods applied. Since the qualitative categorization was performed by a single researcher, the absence of inter-rater validation represents a methodological limitation with regard to reproducibility and potential subjective bias.

Another limitation of this study is the relatively small sample size (*N* = 46), which reduces the stability and generalizability of both the cluster analysis and reliability estimates. While three distinct clusters could be identified, their interpretability and robustness are constrained by the limited number of participants. These clusters are presented here for the first time, providing an initial framework that is exploratory yet grounded in existing literature. Further research with larger, independent samples is needed to validate and more fully characterize these clusters, thereby enhancing the empirical basis of these findings.

The high dropout rate of approximately 59% further exacerbates these limitations, potentially introducing significant non-response bias if those who dropped out differ systematically from the music students who completed the survey. This substantial attrition might reflect issues with survey length, perceived relevance to music students, or the sensitive nature of the short SMI questions, leading to a less representative sample. Similarly, two Short-SMI subscales (Self-Aggrandizer and Bully & Attack) did not reach adequate internal consistency. This may be partly due to the low frequency and socially undesirable nature of these behaviors, which could have led participants to endorse only some items within these scales, resulting in inconsistent responses. Consequently, findings for these subscales should be interpreted cautiously, and future studies with larger, more diverse samples are needed to validate the cluster solution and reliability patterns. Additionally, music students might resemble general student populations more closely than the adult clinical normative samples used in the validation study (Lobbestael et al., 2010), whose mean age was 34 years (SD = 11.80, range 18–70). This pattern may reflect the fact that the phase of life as a student after leaving home is typically characterized by reorientation and upheaval (Slimmen et al., 2022; Worsley et al., 2021). Another limitation of the study is that the exclusion of current diagnoses relied on self-report via the consent form without independent clinical verification. While the comparison study (Lobbestael et al., 2010) investigated clinical and non-clinical samples with specifically assessed selection criteria, this was not possible in the character of an online study, apart from self-reported mental and physical health. While no participants were excluded on this basis, the reliance on self-report may limit the accuracy of diagnostic status in the sample. However, in the case of music students, a heterogeneous field of behavioral and experiential patterns can be assumed, even within the musical disciplines (Nusseck and Spahn, 2013). Furthermore, first-year students at three universities of music were found to have significantly higher values for life satisfaction, social support, and inner peace, as well as for ambition and subjective meaningfulness of work, compared to comparison groups of students of pedagogy and psychology. On the other hand, the ability to distance oneself from work and the striving for perfection were significantly lower than in the comparison groups mentioned (Hildebrandt, 2015).

Another constraint of our study is the limitation of the comparison with clinical samples to DSM-IV and ICD-10 classifications rather than DSM-V and ICD-11 categorization. Axis I refers to clinical disorders such as depression or anxiety, and Axis II refers to personality disorders. While these categories are now outdated in DSM-5/ICD-11, they are retained here because the short SMI validation study used these classifications and provides large, clustered clinical samples that serve as a practical reference for interpreting the schema mode patterns in our music student sample. The classification as Axis I and II is now a historical concept, but was still used at the time of the development of the short SMI (Lobbestael et al., 2010). In DSM-5, the multiaxial system was discontinued to better reflect the dimensional and interacting nature of mental disorders, integrating personality and clinical symptoms within a unified diagnostic framework (American Psychiatric Association and DSM-5 Task Force, 2013). This transition aligns closely with schema theory, which likewise conceptualizes maladaptive modes and coping styles as dimensional phenomena rather than discrete categories. For future research on musicians' health, this conceptual shift implies a move toward dimensional, trait-based profiling of emotional and cognitive patterns—an approach that could bridge music performance psychology with contemporary clinical frameworks. Understanding musicians' schema configurations in a dimensional way could facilitate earlier identification of risk patterns (e.g., perfectionism, rejection sensitivity, or emotional suppression) and guide preventive interventions, coaching, or therapy. As mental health prevention becomes an increasing focus in conservatoires and professional training, adopting updated models consistent with DSM-5 and ICD-11 could enhance both research comparability and the practical translation of psychological findings into evidence-based support for musicians.

Future studies should consider larger samples of cross-university cohorts and include clinical, psychological, and biographical assessments to validate these findings and theoretical considerations. Longitudinal studies could explore the long-term impacts of schema-focused workshops designed for musicians. Additionally, questionnaires that assess the underlying schemas, e.g., YSQ (Young Schema Questionnaire), should be used to identify whether common schemas such as “social isolation” or “rejection” or “failure” are specifically more prevalent in highly competitive populations such as music students and professional musicians or athletes compared to the general student or adult population. This would complete the picture of the related schema modes presented in this research. Additionally, integrating clinical assessments of common physical and psychological diagnoses might offer deeper insights into the interrelation between schema modes and musicians' overall, long-term health, ultimately contributing to their holistic development. Especially the investigation of MPA and musicians' dystonia, as well as psychosomatic components of pain disorders in relation to schemas and schema modes, may inform about the genesis and psychopathology of these disorders and improve individualization of treatment strategies.

### 4.5 Conclusion

This study provides initial evidence that music students exhibit elevated maladaptive schema modes intermediate between non-clinical controls and patients with Axis I disorders, suggesting substantial psychological vulnerabilities related to the demands of professional music training. The exploratory identification of three distinct coping profiles—“Balanced,” “Compensating,” and “Vulnerable Musicians”—suggests that music students may benefit from differentiated, personalized intervention approaches tailored to their specific psychological needs. Qualitative findings further illuminate these vulnerabilities, with students frequently reporting negative self-talk, perceived criticism, and difficulties in teacher–student relationships, underscoring the need for interventions that address both intrapsychic processes and the interpersonal dynamics of music education. The negative associations between maladaptive modes and musician-specific coping indicate that schema-focused interventions targeting emotional regulation, self-criticism, and avoidant coping strategies could potentially enhance students' ability to manage performance demands and sustain career longevity. Music institutions may benefit from implementing comprehensive support systems, including schema-focused workshops, individual coaching with psychologically trained personnel, improved pedagogical training to foster supportive teacher–student relationships, and peer support networks to address the heterogeneous needs identified across coping profiles. However, given the small sample size, high dropout rate, and exploratory nature of these findings, these recommendations remain preliminary. Future research with larger samples, comprehensive clinical assessments, and rigorous intervention studies is essential to validate these profiles and establish the efficacy of schema-based approaches for supporting the psychological wellbeing and professional development of music students.

## Data Availability

The datasets presented in this study can be found in online repositories. The names of the repository/repositories and accession number(s) can be found below: The datasets analyzed for this study can be found in the Zenondo Platform (https://doi.org/10.5281/zenodo.16414979).

## References

[B1] Ackermann B. Driscoll T. Kenny D. T. (2012). Musculoskeletal pain and injury in professional orchestral musicians in Australia. Med. Probl. Perform. Art. 27, 181–187. doi: 10.21091/mppa.2012.4034 23247873

[B2] Alpheis S. Altenmüller E. Scholz D. S. (2022). Influence of adverse childhood experiences and perfectionism on musicians' dystonia: a case control study. Tremor Hyperkinetic Mov. 12:8. doi: 10.5334/tohm.687 PMC8932351 35415008

[B3] Alpheis S. Altenmüller E. Scholz D. S. (2023). “Focal dystonia and the stress network: the role of stress vulnerability and adverse childhood experiences in the development of musicians' dystonia,” in Basic and Translational Applications of the Network Theory for Dystonia, eds. A. Shaikh and A. Sadnicka (Cham: Springer International Publishing), 23–44. doi: 10.1007/978-3-031-26220-3_2 37338694

[B4] Altenmüller E. Jabusch H. C. (2009). Focal hand dystonia in musicians: phenomenology, etiology, and psychological trigger factors. J. Hand Ther. 22, 144–155. doi: 10.1016/j.jht.2008.11.007 19278826

[B5] Altenmüller E. Schlaug G. (2012). Music, brain, and health: exploring biological foundations of music's health effects. Music Health Wellbeing 2, 12–24. doi: 10.1093/acprof:oso/9780199586974.003.0002

[B6] Altenmüller E. Schlaug G. (2013). Neurologic music therapy: the beneficial effects of music making on neurorehabilitation. Acoust. Sci. Technol. 34, 5–12. doi: 10.1250/ast.34.5

[B7] American Psychiatric Association and DSM-5 Task Force. (2013). Diagnostic and statistical manual of mental disorders: DSM-5™*, 5th edn*. American Psychiatric Publishing, Inc. doi: 10.1176/appi.books.9780890425596

[B8] Arntz A. Klokman J. Sieswerda S. (2005). An experimental test of the schema mode model of borderline personality disorder. J. Behav. Ther. Exp. Psychiatry 36, 226–239. doi: 10.1016/j.jbtep.2005.05.005 16004961

[B9] Ascenso S. Williamon A. Perkins R. (2017). Understanding the wellbeing of professional musicians through the lens of positive psychology. Psychol. Music 45, 65–81. doi: 10.1177/0305735616646864

[B10] Bach B. Simonsen E. Christoffersen P. Kriston L. (2015). The Young schema questionnaire 3 short form (YSQ-S3). Eur. J. Psychol. Assess. 33, 134–143. doi: 10.1027/1015-5759/a000272

[B11] Baldassare M. Rosenfield S. Rook K. (1984). The types of social relations predicting elderly well-being. Res. Aging 6, 549–559. doi: 10.1177/0164027584006004006 6544997

[B12] Bamelis L. L. Evers S. M. Spinhoven P. Arntz A. (2014). Results of a multicenter randomized controlled trial of the clinical effectiveness of schema therapy for personality disorders. Am. J. Psychiatry 171, 305–322. doi: 10.1176/appi.ajp.2013.12040518 24322378

[B13] Baník G. Vargová L. Zibrínová L. (2022). Early Maladaptive Schemas, Depression and Post-traumatic Stress Disorder in a Trauma-exposed Sample: A Correlation, Regression and Network Perspective. Center for Open Science. https://osf.io/6fhds (accessed July 9, 2025).

[B14] Basile B. Tenore K. Luppino O. I. Mancini F. (2017). Schema therapy mode model applied to OCD. Clin. Neuropsychiatry 14, 407–414.

[B15] Bernstein D. P. Fink L. Handelsman L. Foote J. (1998). “Childhood trauma questionnaire,” in Assessment of Family Violence: A Handbook for Researchers and Practitioners.

[B16] Borngräber F. Schmidt A. (2020). Neuropsychological aspects of focal dystonia in musicians. Z. Neuropsychol. 31, 69–75. doi: 10.1024/1016-264X/a000293

[B17] Boskovic I. Orthey R. Otgaar H. Mangiulli I. Rassin E. (2023). #StudentsToo. prevalence of sexual assault reports among students of three European universities and their actions post-assault. PLoS ONE 18:e0283554. doi: 10.1371/journal.pone.0283554 37027374 PMC10081757

[B18] Böttcher A. Zarucha A. Köbe T. Gaubert M. Höppner A. Altenstein S. . (2022). Musical activity during life is associated with multi-domain cognitive and brain benefits in older adults. Front. Psychol. 13:945709. doi: 10.3389/fpsyg.2022.945709 36092026 PMC9454948

[B19] Cardoso M. Leonido L. Pereira A. Morgado E. (2025). Mental health challenges in professional musicians: a systematic review of stress, anxiety, and depression. Int. J. Innov. Res. Sci. Stud. 8, 3611–3621. doi: 10.53894/ijirss.v8i2.6065

[B20] Clarke P. Nieuwenhuijsen E. R. (2009). Environments for healthy ageing: a critical review. Maturitas 64, 14–19. doi: 10.1016/j.maturitas.2009.07.011 19695800

[B21] Cloninger C. R. Przybeck T. R. Svrakic D. M. Wetzel R. D. (1994). The Temperament and Character Inventory (TCI): A Guide to Its Development and Use. p. 19–28.

[B22] Csikszentmihalyi M. Csikzentmihaly M. (1990). Flow: The Psychology of Optimal Experience, Vol. 1990. New York, NY: Harper and Row.

[B23] Csikszentmihalyi M. LeFevre J. (1989). Optimal experience in work and leisure. J. Pers. Soc. Psychol. 56:815. doi: 10.1037/0022-3514.56.5.815 2724069

[B24] Curhan A. L. Rabinowitz J. A. Pas E. T. Bradshaw C. P. (2020). Informant discrepancies in internalizing and externalizing symptoms in an at-risk sample: the role of parenting and school engagement. J. Youth Adolesc. 49, 311–322. doi: 10.1007/s10964-019-01107-x 31446584 PMC6992516

[B25] De Jong-Gierveld J. Van Tilburg T. G. (1999). Manual of the Loneliness Scale. Methoden en technieken.

[B26] Deci E. L. Ryan R. M. (2008). Self-determination theory: a macrotheory of human motivation, development, and health. Can. Psychol. Can. 49:182. doi: 10.1037/a0012801

[B27] Deci E. L. Ryan R. M. (2012). Self-determination theory. Handb. Theor. Soc. Psychol. 1, 416–436. doi: 10.4135/9781446249215.n21

[B28] Dews C. L. B. Williams M. S. (1989). Student musicians' personality styles, stresses, and coping patterns. Psychol. Music 17, 37–47. doi: 10.1177/0305735689171004

[B29] Dora G. (2025). Beyond the Music: Narcissism and Fame in Male Rock Musicians. ProQuest. Available online at: https://www.proquest.com/openview/931065ca41253cf4982f46743d92f8ad/1?pq-origsite=gscholarandcbl=18750anddiss=y (accessed October 4, 2025).

[B30] Dreessen L. Arntz A. (1995). The Personality Disorder Beliegs Questionnaire. Maastricht.

[B31] Dutra L. Callahan K. Forman E. Mendelsohn M. Herman J. (2008). Core schemas and suicidality in a chronically traumatized population. J. Nerv. Ment. Dis. 196, 71–74. doi: 10.1097/NMD.0b013e31815fa4c1 18195645

[B32] Elpus K. Carter B. A. (2016). Bullying victimization among music ensemble and theatre students in the United States. J. Res. Music Educ. 64, 322–343. doi: 10.1177/0022429416658642

[B33] Folkman S. Lazarus R. S. Gruen R. J. DeLongis A. (1986). Appraisal, coping, health status, and psychological symptoms. J. Pers. Soc. Psychol. 50:571. doi: 10.1037/0022-3514.50.3.571 3701593

[B34] Fredrickson B. L. (2001). The role of positive emotions in positive psychology: the broaden-and-build theory of positive emotions. Am. Psychol. 56:218. doi: 10.1037/0003-066X.56.3.218 11315248 PMC3122271

[B35] Fredrickson B. L. (2003). The value of positive emotions: the emerging science of positive psychology is coming to understand why it's good to feel good. Am. Sci. 91, 330–335. doi: 10.1511/2003.26.330

[B36] Giesen-Bloo J. Van Dyck R. Spinhoven P. Van Tilburg W. Dirksen C. Van Asselt T. . (2006). Outpatient psychotherapy for borderline personality disorder: randomized trial of schema-focused therapy vs transference-focused psychotherapy. Arch. Gen. Psychiatry 63, 649–658. doi: 10.1001/archpsyc.63.6.649 16754838

[B37] Griffin D. W. Bartholomew K. (1994). “The metaphysics of measurement: the case of adult attachment,” in Advances in Personal Relationships: Attachment Processes in Adult Relationships, Vol. 5, eds. K. Bartholomew and D. Perlman (London: Jessica Kingsley), 17–52.

[B38] Gross E. Stelzer N. Jacob G. (2012). “Treating OCD with the schema mode model,” in The Wiley-Blackwell Handbook of Schema Therapy: Theory, Research, and Practice, eds. M. van Vreeswijk, J. Broersen, and M. Nadort (Wiley), 173–184. doi: 10.1002/9781119962830.ch11

[B39] Harris D. A. (2010). Inhaltliche Validierung der deutschen Übersetzung des “Schema Mode Inventory-revised” (SMI-r) und Korrelation der Modus-Skalen des SMI-r mit etablierten psychologischen und psychopathologischen Konstrukten (Dissertation). University of Freiburg, Breisgau, Germany.

[B40] Hawke L. D. Provencher M. D. (2011). Schema theory and schema therapy in mood and anxiety disorders: a review. J. Cogn. Psychother. 25, 257–276. doi: 10.1891/0889-8391.25.4.257 32759106

[B41] Hildebrandt H. (2004). Musikstudium und Gesundheit. Aufbau und Wirksamkeit eines präventiven Lehrangebotes. Bern: Peter Lang.

[B42] Hildebrandt H. (2009). “Teaching music physiology and motor learning processes at a university: experience and evaluation,” in Art in Motion: Musical and Athletic Motor Learning and Performance, ed. A. Mornell (Frankfurt am Main: Peter Lang), 191–222.

[B43] Hildebrandt H. (2015). “Lampenfieberforschung und musikalischer Berufsalltag,” in Dokumentation zum Zürcher Symposium der SMM ‘Stress und Musizieren Ursachen-Prävention-Bewältigung' (Swissmedmusica), 9–16. Available online at: https://swissmedmusica.ch/books/stress-und-musizieren/ (Accessed July 27, 2025).

[B44] Hildebrandt H. Nübling M. Candia V. (2012). Increment of fatigue, depression, and stage fright during the first year of high-level education in music students. Med. Probl. Perform. Art. 27, 43–48. doi: 10.21091/mppa.2012.1008 22543322

[B45] Ioannou C. I. Altenmüller E. (2014). Psychological characteristics in musician? s dystonia: a new diagnostic classification. Neuropsychologia 61, 80–88. doi: 10.1016/j.neuropsychologia.2014.05.014 24946316

[B46] Ioannou C. I. Furuya S. Altenmüller E. (2016). The impact of stress on motor performance in skilled musicians suffering from focal dystonia: physiological and psychological characteristics. Neuropsychologia 85, 226–236. doi: 10.1016/j.neuropsychologia.2016.03.029 27033741

[B47] Iorwerth M. A. Knox D. (2019). “The application of networked music performance to access ensemble activity for socially isolated musicians,” in Proceedings of the Web Audio Conference 2019–Diversity in Web Audio. Trondheim.

[B48] Iyendo T. O. (2016). Exploring the effect of sound and music on health in hospital settings: a narrative review. Int. J. Nurs. Stud. 63, 82–100. doi: 10.1016/j.ijnurstu.2016.08.008 27611092

[B49] Jabusch H. C. Altenmüller E. (2004). Anxiety as an aggravating factor during onset of focal dystonia in musicians. Med. Probl. Perform. Art. 19, 75–81. doi: 10.21091/mppa.2004.2012 20795373

[B50] Jabusch H. C. Müller S. V. Altenmüller E. (2004). Anxiety in musicians with focal dystonia and those with chronic pain. Mov. Disord. Off. J. Mov. Disord. Soc. 19, 1169–1175. doi: 10.1002/mds.20110 15390020

[B51] Jacob G. A. Arntz A. (2013). Schema therapy for personality disorders—a review. Int. J. Cogn. Ther. 6, 171–185. doi: 10.1521/ijct.2013.6.2.171

[B52] James C. E. Altenmüller E. Kliegel M. Krüger T. H. Van De Ville D. Worschech F. . (2020). Train the brain with music (TBM): brain plasticity and cognitive benefits induced by musical training in elderly people in Germany and Switzerland, a study protocol for an RCT comparing musical instrumental practice to sensitization to music. BMC Geriatr. 20, 1–19. doi: 10.1186/s12877-020-01761-y 33087078 PMC7576734

[B53] Kapsetaki M. E. Easmon C. (2019). Eating disorders in musicians: a survey investigating self-reported eating disorders of musicians. Eat Weight Disord. Stud. Anorex Bulim. Obes. 24, 541–549. doi: 10.1007/s40519-017-0414-9 28710741 PMC6531399

[B54] Karatzias T. Jowett S. Begley A. Deas S. (2016). Early maladaptive schemas in adult survivors of interpersonal trauma: foundations for a cognitive theory of psychopathology. Eur. J. Psychotraumatol. 7. doi: 10.3402/ejpt.v7.30713 27642181 PMC5027329

[B55] Kenny D. (2011). The Psychology of Music Performance Anxiety, 386p. Oxford: OUP. doi: 10.1093/acprof:oso/9780199586141.001.0001

[B56] Kenny D. Ackermann B. (2015). Performance-related musculoskeletal pain, depression and music performance anxiety in professional orchestral musicians: a population study. Psychol. Music 43, 43–60. doi: 10.1177/0305735613493953

[B57] Kenny D. Driscoll T. Ackermann B. (2014). Psychological well-being in professional orchestral musicians in Australia: a descriptive population study. Psychol. Music 42, 210–232. doi: 10.1177/0305735612463950

[B58] Kenny D. T. Ackermann B. J. (2016). “Optimizing physical and psychological health in performing musicians,” in The Oxford Handbook of Music Psychology, eds. S. Hallam, I. Cross, and M. H. Thaut (Oxford: Oxford University Press), 633–648.

[B59] Korte M. Cerci D. Wehry R. Timmers R. Williamson V. J. (2025). The patient musician: a qualitative investigation of professional classical musicians who previously suffered from depression. BMC Public Health 25, 1–10. doi: 10.1186/s12889-025-22656-w 40312314 PMC12044710

[B60] Kreutz G. Bongard S. Rohrmann S. Hodapp V. Grebe D. (2004). Effects of choir singing or listening on secretory immunoglobulin A, cortisol, and emotional state. J. Behav. Med. 27, 623–635. doi: 10.1007/s10865-004-0006-9 15669447

[B61] Kreutz G. Murcia C. Q. Bongard S. (2012). Psychoneuroendocrine research on music and health: an overview. Music Health Wellbeing 27, 457–476. doi: 10.1093/acprof:oso/9780199586974.003.0030

[B62] Kriston L. Schäfer J. Jacob G. A. Härter M. Hölzel L. P. (2013). Reliability and validity of the German version of the Young schema questionnaire–short form 3 (YSQ-S3). Eur. J. Psychol. Assess. 29, 205–212. doi: 10.1027/1015-5759/a000143

[B63] Kuhn D. (2002). The effects of active and passive participation in musical activity on the immune system as measured by salivary immunoglobulin A (SlgA). J. Music Ther. 39, 30–39. doi: 10.1093/jmt/39.1.30 12015810

[B64] Lazarus R. S. Folkman S. (1984). Stress, Appraisal, and Coping. New York, NY: Springer Publishing Company.

[B65] Leaver R. Harris E. C. Palmer K. T. (2011). Musculoskeletal pain in elite professional musicians from British symphony orchestras. Occup. Med. 61, 549–555. doi: 10.1093/occmed/kqr129 22003061 PMC3428866

[B66] Lobbestael J. Arntz A. Sieswerda S. (2005). Schema modes and childhood abuse in borderline and antisocial personality disorders. J. Behav. Ther. Exp. Psychiatry 36, 240–253. doi: 10.1016/j.jbtep.2005.05.006 15953584

[B67] Lobbestael J. van Vreeswijk M. Arntz A. (2007). Netherlands Journal of Psychology, 2007, number 3 Shedding light on schema modes: a clarification of the mode concept and its current research status. Neth. J. Psychol. 63, 69–78. doi: 10.1007/BF03061068

[B68] Lobbestael J. Van Vreeswijk M. Spinhoven P. Schouten E. Arntz A. (2010). Reliability and validity of the short schema mode inventory (SMI). Behav. Cogn. Psychother. 38, 437–458. doi: 10.1017/S1352465810000226 20487590

[B69] Lobbestael J. Van Vreeswijk M. F. Arntz A. (2008). An empirical test of schema mode conceptualizations in personality disorders. Behav. Res. Ther. 46, 854–860. doi: 10.1016/j.brat.2008.03.006 18460405

[B70] Loveday C. Musgrave G. Gross S. A. (2023). Predicting anxiety, depression, and wellbeing in professional and nonprofessional musicians. Psychol. Music 51, 508–522. doi: 10.1177/03057356221096506

[B71] Masley S. A. Gillanders D. T. Simpson S. G. Taylor M. A. (2012). A systematic review of the evidence base for schema therapy. Cogn. Behav. Ther. 41, 185–202. doi: 10.1080/16506073.2011.614274 22074317

[B72] McCormick I. A. Passmore J. Leach S. (2022). “Schema coaching: theory, research and practice,” in Third *Wave Cognitive Behavioural Coaching: Contextual, Behavioural and Neuroscience Approaches for Evidence-Based Coaches*, eds. P. Jonathan and S. Leach (Shoreham-by-Sea: Pavilion Publishing and Media Ltd), 173–193.

[B73] Middlestadt S. E. Fishbein M. (1988). Health and occupational correlates of perceived occupational stress in symphony orchestra musicians. J. Occup. Med. 30, 687–692. 3183784

[B74] Migge B. (2014). Handbuch Coaching und Beratung: wirkungsvolle Modelle, kommentierte Falldarstellungen, zahlreiche Übungen; mit umfangreichen Downloadmaterialien. Weinheim: Beltz.

[B75] Moreno S. Marques C. Santos A. Santos M. Castro S. L. Besson M. . (2009). Musical training influences linguistic abilities in 8-year-old children: more evidence for brain plasticity. Cereb. Cortex 19, 712–723. doi: 10.1093/cercor/bhn120 18832336

[B76] Münte T. F. Altenmüller E. Jäncke L. (2002). The musician's brain as a model of neuroplasticity. Nat. Rev. Neurosci. 3, 473–478. doi: 10.1038/nrn843 12042882

[B77] Musgrave G. (2023a). Musicians, their relationships, and their wellbeing: creative labour, relational work. Poetics 96:101762. doi: 10.1016/j.poetic.2023.101762

[B78] Musgrave G. (2023b). Music and wellbeing vs. musicians' wellbeing: examining the paradox of music-making positively impacting wellbeing, but musicians suffering from poor mental health. Cult. Trends 32, 280–295. doi: 10.1080/09548963.2022.2058354

[B79] Nagel J. J. (1990). Performance anxiety and the performing musician: a fear of failure or a fear of success? Med. Probl. Perform. Art. 5, 37–40.

[B80] Newman C. George R. P. Beitz T. Bergson Z. Zemon V. (2022). Mental health issues among international touring professionals in the music industry. J. Psychiatr. Res. 145, 243–249. doi: 10.1016/j.jpsychires.2021.12.031 34942435

[B81] Nunnally J. Bernstein I. (1994). Psychometric Theory, 3rd Edn. New York, NY: MacGraw-Hill.

[B82] Nusseck M. Spahn C. (2013). Vergleich der studienbezogenen Verhaltens-und Erlebensmuster bei Musikstudierenden des künstlerischen Hauptfaches und der Schulmusik. Musik Musik 20, 117–125.

[B83] Özevin B. (2022). Music class and abuse. Athens J. Educ. 9, 575–592. doi: 10.30958/aje.9-4-3

[B84] Peeters N. van Passel B. Krans J. (2022). The effectiveness of schema therapy for patients with anxiety disorders, OCD, or PTSD: a systematic review and research agenda. Br. J. Clin. Psychol. 61, 579–597. doi: 10.1111/bjc.12324 34296767 PMC9544733

[B85] Penney E. S. Norton A. R. (2022). A novel application of the schema therapy mode model for social anxiety disorder: a naturalistic case study. Clin. Case Stud. 21, 34–47. doi: 10.1177/15346501211027866

[B86] Phillips K. Brockman R. Bailey P. E. Kneebone I. I. (2019). Young schema questionnaire–short form version 3 (YSQ-S3): preliminary validation in older adults. Aging Ment. Health 23, 140–147. doi: 10.1080/13607863.2017.1396579 29125326

[B87] Price J. P. (2007). Cognitive schemas, defence mechanisms and post-traumatic stress symptomatology. Psychol. Psychother. Theory Res. Pract. 80, 343–353. doi: 10.1348/147608306X144178 17877860

[B88] Raeburn S. D. (2000). Psychological issues and treatment strategies in popular musicians: a review, part 2. Med. Probl. Perform. Art. 15, 6–16. doi: 10.21091/mppa.2000.1003

[B89] Ramstedt A. (2023). Emotional abuse in classical music education in Finland: a study of Finnish women musicians' experiences. Action Crit. Theory Music Educ. 22, 198–226. doi: 10.22176/act22.3.198

[B90] Reis H. T. Gable S. L. (2003). “Toward a positive psychology of relationships,” in Flourishing: Positive Psychology and the Life Well-Lived, eds. C. L. M. Keyes and J. Haidt (American Psychological Association), 129–159. doi: 10.1037/10594-006

[B91] Renner F. Arntz A. Leeuw I. Huibers M. (2013). Treatment for chronic depression using schema therapy. Clin. Psychol. Sci. Pract. 20:166. doi: 10.1111/cpsp.12032

[B92] Reybrouck M. Vuust P. Brattico E. (2018). Music and brain plasticity: how sounds trigger neurogenerative adaptations. Neuroplast. Insights Neural Reorg. 85, 85–103. doi: 10.5772/intechopen.74318

[B93] Rezaei M. Ghazanfari F. Rezaee F. (2016). The role of childhood trauma, early maladaptive schemas, emotional schemas and experimental avoidance on depression: a structural equation modeling. Psychiatry Res. 246, 407–414. doi: 10.1016/j.psychres.2016.10.037 27788461

[B94] Rijkeboer M. (2012). “Validation of the young schema questionnaire,” in The Wiley-Blackwell Handbook of Schema Therapy: Theory, Research, and Practice, eds. M. van Vreeswijk, J. Broersen, and M. Nadort (Wiley), 531–539. doi: 10.1002/9781119962830.ch40

[B95] Robinson M. Doherty D. A. Cannon J. Hickey M. Rosenthal S. L. Marino J. L. . (2019). Comparing adolescent and parent reports of externalizing problems: a longitudinal population-based study. Br. J. Dev. Psychol. 37, 247–268. doi: 10.1111/bjdp.12270 30394545

[B96] Ryan R. M. Deci E. L. (2008). A self-determination theory approach to psychotherapy: the motivational basis for effective change. Can. Psychol. Can. 49:186. doi: 10.1037/a0012753

[B97] Ryan R. M. Patrick H. Deci E. L. Williams G. C. (2008). Facilitating health behaviour change and its maintenance: interventions based on self-determination theory. Eur. Health Psychol. 10, 2–5.

[B98] Sataloff R. T. Brandfonbrener A. G. Lederman R. J. (1998). Performing Arts Medicine. Norwich: Singular Publishing Group.

[B99] Schneider J. Scholz D. S. Altenmüller E. (2021). Impact of psychic traumatization on the development of musicians' dystonia: six exploratory case studies. Med. Probl. Perform. Art. 36, 1–9. doi: 10.21091/mppa.2021.1001 33647091

[B100] Schreurs P. J. G. van de Willige G. Brosschot J. F. (1993). De Utrechtse Coping Lijst: UCL [The Utrecht Coping List]. Lisse: Swets en Zeitlinger.

[B101] Sihvonen A. J. Särkämö T. Leo V. Tervaniemi M. Altenmüller E. Soinila S. . (2017). Music-based interventions in neurological rehabilitation. Lancet Neurol. 16, 648–660. doi: 10.1016/S1474-4422(17)30168-0 28663005

[B102] Silva A. G. Lã F. Afreixo V. (2015). Pain prevalence in instrumental musicians: a systematic review. Med. Probl. Perform. Art. 30, 8–19. doi: 10.21091/mppa.2015.1002 25743601

[B103] Simpson S. G. Pietrabissa G. Rossi A. Seychell T. Manzoni G. M. Munro C. . (2018). Factorial structure and preliminary validation of the schema mode inventory for eating disorders (SMI-ED). Front. Psychol. 9:600. doi: 10.3389/fpsyg.2018.00600 29740379 PMC5928750

[B104] Skaggs R. (2019). Socializing rejection and failure in artistic occupational communities. Work Occup. 46, 149–175. doi: 10.1177/0730888418796546

[B105] Slimmen S. Timmermans O. Mikolajczak-Degrauwe K. Oenema A. (2022). How stress-related factors affect mental wellbeing of university students a cross-sectional study to explore the associations between stressors, perceived stress, and mental wellbeing. PLoS ONE 17:e0275925. doi: 10.1371/journal.pone.0275925 36342914 PMC9639818

[B106] Sokoli E. Hildebrandt H. Gomez P. (2022). Classical music students' pre-performance anxiety, catastrophizing, and bodily complaints vary by age, gender, and instrument and predict self-rated performance quality. Front. Psychol. 13:905680. doi: 10.3389/fpsyg.2022.905680 35814093 PMC9263585

[B107] Spadine M. Patterson M. S. Brown S. Nelon J. Lanning B. Johnson D. M. . (2022). Predicting emotional abuse among a sample of college students. J. Am. Coll. Health 70, 256–264. doi: 10.1080/07448481.2020.1740709 32208068

[B108] Spahn C. (2018). Psychosomatische und psychische Erkrankungen bei Musikern. Nervenheilkunde 37, 398–408. doi: 10.1055/s-0038-1667396

[B109] Spahn C. Krampe F. Nusseck M. (2021a). Live music performance: the relationship between flow and music performance anxiety. Front. Psychol. 12:725569. doi: 10.3389/fpsyg.2021.725569 34899468 PMC8655696

[B110] Spahn C. Krampe F. Nusseck M. (2021b). Classifying different types of music performance anxiety. Front. Psychol. 12:538535. doi: 10.3389/fpsyg.2021.538535 33967870 PMC8102674

[B111] Spahn C. Walther J. C. Nusseck M. (2016). The effectiveness of a multimodal concept of audition training for music students in coping with music performance anxiety. Psychol. Music 44, 893–909. doi: 10.1177/0305735615597484

[B112] Spielberger C. D. Jacobs G. A. Russel S. F. Crane R. S. (1983). “Assessment of anger: the State Trait Anger Scale,” in Advances in Personality Assessment, Vol. 2, eds. J. Butcher and C. Spielberger (Hillsdale, NJ: Erlblaum), 112–134.

[B113] Spintge R. Droh R. (2012). Musik in der medizin/music in medicine: neurophysiologische grundlagen klinische applikationen geisteswissenschaftliche einordnung/neurophysiological basis clinical applications aspects in the humanities. Springer Science and Business Media.

[B114] Steele B. Martin M. Sciarra A. Melendez-Torres G. J. Degli Esposti M. Humphreys D. K. . (2024). The prevalence of sexual assault among higher education students: a systematic review with meta-analyses. Trauma Violence Abuse 25, 1885–1898. doi: 10.1177/15248380231196119 37728132 PMC11155219

[B115] Steyn B. J. Steyn M. H. Maree D. J. Panebianco-Warrens C. (2016). Psychological skills and mindfulness training effects on the psychological wellbeing of undergraduate music students: an exploratory study. J. Psychol. Afr. 26, 167–171. doi: 10.1080/14330237.2016.1163906

[B116] Sullivan G. M. Feinn R. (2012). Using effect size—or why the *P* value is not enough. J. Grad. Med. Educ. 4, 279–282. doi: 10.4300/JGME-D-12-00156.1 23997866 PMC3444174

[B117] Swart I. (2016). Ego boundaries and self-esteem: two elusive facets of the psyche of performing musicians. Psychol. Music 44, 691–709. doi: 10.1177/0305735615590283

[B118] Talbot D. Smith E. Tomkins A. Brockman R. Simpson S. (2015). Schema modes in eating disorders compared to a community sample. J. Eat Disord. 3, 1–4. doi: 10.1186/s40337-015-0082-y 26594359 PMC4653836

[B119] Tenore K. Mancini F. Basile B. (2018). Schemas, modes and coping strategies in obsessive-compulsive like symptoms. Clin. Neuropsychiatry 15, 384–392.

[B120] Thoma M. V. La Marca R. Brönnimann R. Finkel L. Ehlert U. Nater U. M. . (2013). The effect of music on the human stress response. PLoS ONE 8:e70156. doi: 10.1371/journal.pone.0070156 23940541 PMC3734071

[B121] Timmerman I. Sanderman R. Koopmans P. C. Emmelkamp P. M. G. (1993). Het meten van irrationele cognities met de Irrational Belief Inventory (IBI-50): een handleiding. Groningen: Noordelijk Centrum voor Gezondheitsvraagstukken (NCG); Irrational Beliefs Inventory (IBI).

[B122] Vaag J. Bjørngaard J. H. Bjerkeset O. (2016a). Symptoms of anxiety and depression among Norwegian musicians compared to the general workforce. Psychol. Music 44, 234–248. doi: 10.1177/0305735614564910

[B123] Vaag J. Bjørngaard J. H. Bjerkeset O. (2016b). Use of psychotherapy and psychotropic medication among Norwegian musicians compared to the general workforce. Psychol. Music 44, 1439–1453. doi: 10.1177/0305735616637132

[B124] Vaag J. Saksvik-Lehouillier I. Bjørngaard J. H. Bjerkeset O. (2016c). Sleep difficulties and insomnia symptoms in Norwegian musicians compared to the general population and workforce. Behav. Sleep Med. 14, 325–342. doi: 10.1080/15402002.2015.1007991 26337077

[B125] Vervainioti A. Alexopoulos E. (2015). Job-related stressors of classical instrumental musicians: a systematic qualitative review. Med. Probl. Perform. Art. 30, 197–202. doi: 10.21091/mppa.2015.4037 26614973

[B126] Wan C. Y. Schlaug G. (2010). Music making as a tool for promoting brain plasticity across the life span. Neuroscientist 16, 566–577. doi: 10.1177/1073858410377805 20889966 PMC2996135

[B127] Wenhart T. (2024). Mental Stark, psychisch gesund - Konzeption von Schema-Workshops für Musiker:innen und Musiklehrkräfte. Available online at: https://rgdoi.net/10.13140/RG.2.2.21773.51686 (accessed June 18, 2025).

[B128] Worschech F. Marie D. Sinke C. Kliegel M. Jünemann K. Scholz D. S. . (2025). Quality of life in older adults is enhanced by piano practice: results from a randomized controlled trial. Ann. N.Y. Acad. Sci. 1550, 239–254. doi: 10.1111/nyas.15397 40571975 PMC12412713

[B129] Worsley J. D. Harrison P. Corcoran R. (2021). Bridging the gap: exploring the unique transition from home, school or college into university. Front. Public Health 9:634285. doi: 10.3389/fpubh.2021.634285 33816421 PMC8009977

[B130] Young J. E. Arntz A. Atkinson T. Lobbestael J. Weishaar M. E. van Vreeswijk M. F. . (2007). The Schema Mode Inventory. New York, NY: Schema Therapy Institute.

[B131] Young J. E. Klosko J. S. Weishaar M. E. (2006). Schema Therapy: A Practitioner's Guide. New York, NY: Guilford Press.

