# Supplementary material for: Music students' psychological profiles: unveiling three coping clusters using schema mode inventory
Source: Front Psychol. 2026 Jan 5;16:1673100. doi: 10.3389/fpsyg.2025.1673100 (PMC12812958; doi:10.3389/fpsyg.2025.1673100)
Supplement: Supplementary file 1 [file Data_Sheet_1.pdf]

Supplementary Table 1. Summary of HIL comparison studies.

Sample	Sample size	Study Design	HIL Sum Score (SD)		Source
			Timepoint 1	Timepoint 2	
First Year Music University Students	N=38	Longitudinal study, pre-post design (first study year)	33.33 (4.39)	33.06 (4.62)	Hildebrandt, H. (2004)
First Year Music University Students	N=105	Longitudinal study, pre-post design (first study year)	31.7 (0.45)	31.9 (0.45)	Hildebrandt, H., Nübling, M., & Candia V. (2012)
Music University Students from all semesters	N=46	Controlled intervention study (pre-post design)	intervention group:<br>24.6 (5.7)<br>control group:<br>30.7 (5.2)	intervention group:<br>28.7 (5.4)<br>control group:<br>29.6 (5.9)	Hildebrandt, H. (2004)
Music School Teachers	N=22	Intervention study (pre-post design)	26.8 (5.16)	30.0 (4.78)	Hildebrandt, H. (2004)

Supplementary Table 2. Coding scheme for qualitative analysis. Categories are defined with illustrative anonymized quotes from the dataset.

	**Category**	**Definition / Description**	**Example Quote(s) (anonymized)**
**If you have complaints related to making music, what are they?**<br>**(Question 1)**	Pain	Physical discomfort experienced during practice or performance, including aches or persistent pain in muscles or joints.	"Sometimes pain while playing"<br>"Physical pain (Arms, back)"

	Self-criticism/ Own expectations/Comparison with others	Internal evaluation of one's own performance, often benchmarked against personal standards or the perceived performance of peers.	"a lot of self-critic, sometimes feeling overwhelmed by the process or feeling that I actually don't know what I am doing"<br>"Comparison with others"
	Injuries	Specific physical impairments caused by practice or performance, such as tendonitis, inflammation, or repetitive strain injuries.	"inflammation"<br>"I've had tendonitis"
	Tension/Physical Discomfort	Muscular or bodily tension affecting posture, breathing, or movement during practice or performance, not necessarily associated with injury.	"Wrong usage of muscles. Meaning that my body feels sore sometimes."<br>"Sometimes I have physical discomforts such as hand problems or tensions in parts of my body"
	Confidence on stage/Stage Fright	Feelings of self-assurance or anxiety when performing publicly.	"not being able to do everything as confident as I practiced"<br>"I get very nervous in stage"
	Work-Life-Balance/Self-organization	Ability to manage time, commitments, and personal life alongside practice and performance responsibilities.	"Finding a balance between the work I put in, the projects I choose to do and having time to recover and do other activities and time off (non-related to music) without feeling bad about it"
	Expectations of others	Perceived or actual pressures from teachers, peers, audiences, or family regarding performance quality or behavior.	"not the level they want"
	Dysphonia	Vocal strain, voice loss, or difficulties in vocal production impacting musical performance.	"dysphonia (due to harmful vocal habits) in the past"<br>"voice loss"
	Social conflicts	Disagreements or interpersonal tension within the musical or educational environment	"People have to deliberately behave in an orchestra"

**If you have/have had problems with a teacher or orchestra or chamber music partner currently or in the past, what problems were they?**<br>**(Question2)**	Communication	Effectiveness of verbal or nonverbal interactions with teachers, peers, or ensemble members.	“bad communication”<br>“Authoritarian conductors, music partners with whom it's difficult to communicate”<br>“Not same point of view, communication”
	Lack of quality of teaching (pedagogical method, technique, inadequate feedback)	Perception that instruction is ineffective, unclear, or insufficiently supportive of skill development.	“I didn't feel very supported with her style of teaching.”<br>“During lessons I was sometimes not given enough time to try implementing Feedback in my playing. The result was frustration on both sides and a feeling of not being able to implement Feedback in general.”<br>“Teacher has no pedagogical education”<br>“a former teacher that used giving me bad marks and comments as a "motivation" method (it caused me to feel pressured to practice but not wanting to)”
	Social interaction & organization in ensemble	Quality and structure of collaboration, coordination, and interpersonal relationships within a musical group.	“I was questioned by stand partner of not prepared for the music in a very annoyed temper, while he himself played wrong notes and missed entrances as well. I kindly reminded the previous stand of wrong bowings after their many playthroughs, making her totally annoyed and rejecting to listen.”<br>“Not respecting the rehearsal schedule”
	Distress due to the mood of teacher	Emotional impact of perceived negative affect, frustration, or stress expressed by teachers during lessons or rehearsals.	“I had issues with my past two teachers. They were very jealous when I left for another teacher.”<br>“The quality of the lesson was related to her mood, why I never knew if the lessons were going to be good or not.”
	Lack of respect/sexism/discrimination	Experiences of unfair treatment, bias, or disrespect based on gender, identity, or other personal attributes.	“sexist atmospheres in which as a woman you feel dismissed or ignored”<br>“Teachers that treat students without respect, verbal abuse”

	Anxiety/Stress to perform	Psychological tension, worry, or nervousness related to upcoming or ongoing performance situations.	“I am making stupid mistakes (notes, rythm) because of stress.”
**What are typical thoughts (positive and negative) you have in musical situations (practice, rehearsal, lesson, stage)?** **(Question 3)**	Joy/Pleasure	Positive emotions derived from music-making, practice, or performance.	“On a good day I very much enjoy feeling and expressing the music that I am playing”
	Goal-orientation	Focus on achieving specific musical or personal objectives, including technical or artistic milestones.	“Come on, if you stick with this piece for ten more minutes, you get to enjoy the next piece!” “That is a method I want to try out during practicing!”
	Realistic Self-reflection/Self-Structuring	Ability to objectively evaluate performance and plan practice effectively to improve skills.	“I am ready to display my musicianship even despite some shortcomings of technique, I can play beautifully, I am good at playing with other people and enjoy it.” “I take everything as a lesson and take notes from them, aiming for a better appearance next time.”
	Connection with others/Belonging	Feelings of inclusion, social support, or shared musical identity within ensembles or learning environments.	“Pleasure to connect with different people and grateful to be able to do so” “I love making music and sharing it with my colleagues and public.”
	Gratefulness	Appreciation for opportunities, support, learning experiences, or musical achievements.	“It's a privilege that I can do it” “Pleasure to connect with different people and grateful to be able to do so”
	Flow	State of deep engagement and immersion in practice or performance, often characterized by effortless concentration.	“feeling of being really focused / in the music while practising or during a concert” “expressing the music that I am playing and I can really seize the moment”
	Process-orientation	Focus on learning, technique, and development rather than solely on outcomes or achievements.	“I think about the notes, the way I played and try to improve it.” “listening to others and myself, observing”

	Curiosity/ Surprise/Excitement	Experiencing interest, novelty, or creative stimulation in music-making or learning.	"Excitement over a new piece."<br>"Pleasure to discover new music"
	Confidence /Pride	Positive self-assessment of ability or achievement, leading to feelings of accomplishment and self-worth.	"If I practice with this specific technique, I will be able to play the piece within the next week!"<br>"I can make it through this, I have practiced very well!"
	Imagination	Use of creativity and mental visualization in musical interpretation, practice, or performance	"imagining applause after successful concert"<br>"I sometimes make a plan of what to think when during the piece, such as "forte" or "clouds" or "heavy feet""
	Self-doubt	Uncertainty or lack of trust in one's own musical abilities or decisions.	"In many musical situations I can feel a lot of self doubt"<br>"There's no way I'm learning this by heart"
	Feeling of Inferiority/Insufficiency	Sense of not measuring up to standards, peers, or expectations.	"my teacher will be disappointed"<br>"I think sometimes that I know nothing compared to others."
	Opinion of/Comparison with others	Judging one's own performance based on perceived evaluation of peers, teachers, or audiences.	"everyone will think I'm playing really bad"<br>"Do other people think I should have chosen a different instrument/profession"<br>"I hope no one think that I'm playing to bad to be here"
	Demanding self-critic	Excessively harsh self-evaluation and high expectations for performance or practice outcomes.	"Why didn't I find enough time to practice"<br>"I pressure myself a lot."
	Fear/Nervousness	Acute anxiety or apprehension related to upcoming or ongoing performance, practice, or evaluation.	"Worried to disappoint or not be able to show what I want in a performance situation due to stress"<br>"I will sound amateur, I will mess up intonation etc."
	Hyperfocus on body or technique/errors	Excessive attention to physical execution, technical details, or mistakes, often disrupting musical flow.	"I'm scared that I have no breathing anymore."<br>"my bowhold is weird, I can't even bow straight, shit. I make weird movements with my head / face"

			“Why do my hands feel so heavy/slow/cramped?”
	Shame/Not belonging	Negative self-consciousness or perception of exclusion in musical or educational contexts.	“I feel ashamed that I am not as far/as good as them”<br>“Fear of letting people see what happens when I'm nervous”
	Punishing self-critic/self-hate	Severe internal criticism leading to feelings of self-rejection or emotional distress.	“I'm a bad musician”<br>“I hate this sound. How come I can't even produce a decent sound?”<br>“I am not even capable of practicing, who am I fooling?”
	Frustration/Annoyance	Emotional response to obstacles, mistakes, or perceived slow progress in practice or performance.	“Frustration over a passage not sounding how i would like it to.”<br>“annoyed that there is no focus”<br>“Lots of times I am oriented on results, and if they do not balance with the invested time, I get frustrated.”
	Hopelessness /Disappointment	Sense of failure, stagnation, or inability to achieve desired musical outcomes.	“having the feeling pieces don't improve even though I practise a lot”<br>“I will never be able to work because I need a minimum level of technique to be asked for gigs and I am way below that level.”
	Fear of future/not good enough	Worry or anxiety about long-term prospects in musical development, performance career, or skill attainment.	“I will never make it because I do not know how to work”<br>“I'm not good enough to be a professional”<br>“is it enough to make a living out of it”
